# Nonlinear stiffness of NompC gating spring and its implication in mechanotransduction

**DOI:** 10.1126/sciadv.aeb6165

**Published:** 2026-04-01

**Authors:** Yukun Wang, Peng Jin, Avinash Kumar, Lily Jan, Yifan Cheng, Yuh-Nung Jan, Yongli Zhang

**Affiliations:** ^1^ Department of Cell Biology, Yale University School of Medicine, New Haven, CT, USA.; ^2^ Department of Physiology, University of California, San Francisco, CA, USA.; ^3^ Department of Biochemistry and Biophysics, University of California, San Francisco, CA, USA.; ^4^ Howard Hughes Medical Institute, UCSF, San Francisco, CA, USA.; ^5^ Department of Molecular Biophysics and Biochemistry, Yale University, New Haven, CT, USA.

## Abstract

Cytoskeleton-tethered mechanosensitive channels (MSCs) use compliant gating springs to convert mechanical stimuli into electrical signals for sensations like sound and touch. The mechanical properties of these gating springs are poorly understood. We investigated the homotetrameric NompC channel, which contains long ankyrin-repeat domains (ARDs), using a toehold-mediated strand displacement method to tether single membrane proteins. This method allowed precise force application and extension measurement with optical tweezers. Our results show that a single NompC complex has a low stiffness of ~0.7 piconewtons per nanometer when pulled from one ARD, with stepwise unfolding beginning at ~7 piconewtons, leading to nonlinear stiffness. ARD truncation indicates strong lateral interactions between ARDs. Computational analyses suggest that this nonlinear, low stiffness may regulate NompC’s sensitivity, dynamic range, and kinetics in detecting mechanical stimuli. Our findings highlight the role of a compliant, unfolding-refolding gating spring in facilitating a graded response in MSC ion transduction across diverse mechanical stimuli.

## Teaser

The ankyrin-repeat domains of the NompC complex are flexible and prone to unfolding, enabling regulation of mechanotransduction.

## INTRODUCTION

**Fig. 1.**
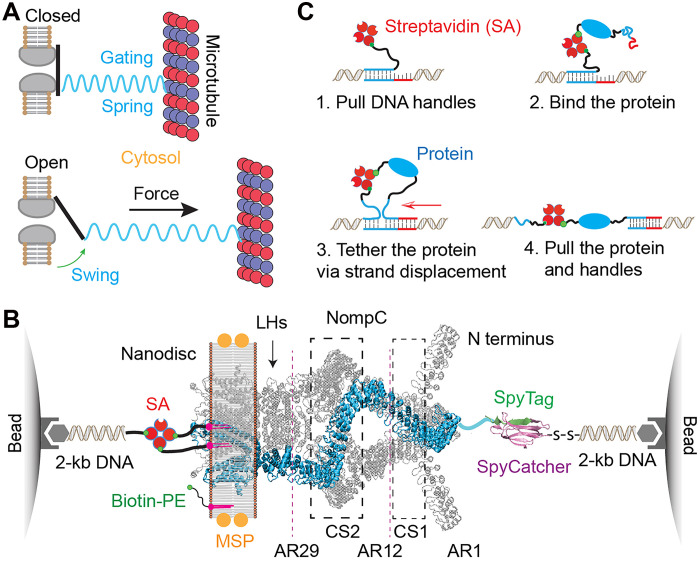
Toehold-mediated strand displacement strategy to measure the elasticity and unfolding of a single NompC complex using high-resolution optical tweezers. (**A**) Schematic diagram showing the channel-opening transition of NompC induced by the force exerted by the microtubule and transmitted by the gating spring. (**B**) Experimental setup to pull a single NompC homotetramer embedded in a lipid nanodisc (Protein Data Bank ID 5VKQ). The NompC complex was tethered between two 2.1-μm beads via two DNA handles through biotin-streptavidin (SA) and SpyTag-SpyCatcher interactions. The positions of the ankyrin repeats (AR1-29), the two ARD contact sites CS1 (AR7-12) and CS2 (AR16-26), and the linker helices (LHs) are indicated. MSP, membrane scaffolding protein. (**C**) Steps to link a single protein to two prestretched DNA handles via toehold-mediated strand displacement and pull the protein using dual-trap optical tweezers (see also movie S1).

Mechanosensitive ion channels (MSCs) detect mechanical forces acting on cell membranes, arising from stimuli such as touch, sound, blood pressure, osmotic pressure, and cell motion (*1*, *2*). While most MSCs sense force within the membrane or through membrane tension, some are tethered to extracellular or intracellular structures via flexible filaments or gating springs, detecting forces external to the membrane (Fig. 1A) (*3*, *4*). A prominent example is the MSC in hair cells of the inner ear essential for hearing (*5*, *6*). However, the mechanisms by which mechanical force gates these tethered MSCs remain poorly understood, partly because of challenges in reconstituting MSC systems in vitro and applying precise forces (*6*, *7*). NompC, a member of the transient receptor potential family, exemplifies tethered MSCs (*3*). It is found in sensory organs across organisms from worms to lower vertebrates, mediating sound, touch, and proprioception (*8*–*10*). NompC is a homotetramer, with each subunit containing an N-terminal, likely disordered polypeptide (123 amino acids) that presumably mediates NompC binding to microtubules, a long ankyrin-repeat domain (ARD; 1013 amino acids), a small helical linker domain called linker helices (127 amino acids), and a pore-forming domain (Fig. 1B) (*9*, *11*). Previous studies indicate that the ARD plays a pivotal role in transmitting force from the cytoskeleton to the channel (*9*), yet the mechanisms of force transmission and gating remain elusive.

Gating springs transmit forces from various mechanical stimuli to channel domains, making their mechanical properties critical for mechanosensation in tethered MSCs (*12*). These springs may experience substantial forces even in a resting cell state and large force changes in response to mechanical stimuli, requiring mechanical stability (*13*). Stimuli manifest as displacements of membranes, cytoskeleton, hair bundles, or other sensors, ranging from subnanometers to hundreds of nanometers (*14*). To transduce such a wide range of displacements into typically nanometer-scale conformational changes in the channel, a flexible gating spring is essential for a graded current response. Moreover, the stiffness and stability of the gating spring must be regulated to balance sensitivity and dynamic range (*13*). Sensitivity to small displacements requires high stiffness, while large displacements necessitate low stiffness or protein unfolding (*15*), suggesting that gating spring stiffness is nonlinear with force.

The stiffness and mechanical stability of ankyrin repeats (ARs) are crucial for their function but remain poorly understood. The 33–amino acid AR motif is highly abundant, appearing in tandem repeats in more than 400 human proteins (*16*). Many ARDs link the cytoskeleton to diverse membrane targets. Howard and Bechstedt (*17*) proposed that NompC ARDs act as flexible gating springs, estimating a stiffness of ~1 pN/nm per ARD based on their helical structure and the shear modulus of rigid proteins. Similarly, the 24 ARDs in the ankyrin repeat B protein (AnkB) may contribute to the gating spring of *Caenorhabditis elegans* TMC1 (*6*), whose mammalian homologs TMC1/2 are implicated in hearing (*6*, *18*). However, atomic force microscopy (AFM) suggests that the AnkB ARD is stiffer (>1.9 pN/nm) (*19*, *20*), and molecular dynamics simulations predict even higher stiffness (4 to 5 pN/nm for the AnkB ARD, 4 pN/nm for a single NompC ARD, or 13 pN/nm for the tetramer) (*21*–*23*). AFM also indicates that AnkB ARD is mechanically stable, unfolding above 20 pN at high pulling rates (>100 pN/s) (*19*, *20*), while NompC ARD stability remains unmeasured. Accordingly, a recent study suggests that NompC linker helices, instead of ARDs, may serve as the gating spring for the NompC channel (*24*). These conflicting results necessitate a better understanding of the mechanical properties of ARDs and their roles in MSC gating.

The force transmitted by gating springs may trigger discrete conformational changes in tethered MSCs to open their channels, accompanied by energy increases (*14*). These energy changes are offset by mechanical work from the gating force, requiring corresponding extension changes (gating swing) along the force direction (Fig. 1A). Thus, gating swing and force are key parameters of tethered MSCs, critical for their sensitivity and dynamic range. For mechanotransduction channels in vertebrate hair cells, the gating swing is estimated at 2 to 6 nm (*25*), with gating forces ranging from 5 to 35 pN, depending on the cell type and cochlear location (*13*). However, these parameters are unreported for NompC.

To address these questions, we developed a toehold-mediated strand displacement strategy to measure both monotonic and discrete extension changes of a single nanodisc-anchored NompC complex using high-resolution optical tweezers (*26*, *27*). We characterized the mechanical properties of single NompC complexes, finding that individual ARDs are compliant and unfold at low forces, potentially facilitating mechanical stimulus transmission to ion transduction. We also monitored a conformational change possibly linked to NompC’s gating transition. Our results support the role of the ARDs as gating springs and illuminate NompC’s gating mechanism.

## RESULTS

### A differential approach for measuring the absolute extension of a single-protein complex using optical tweezers

Optical tweezers are widely used to study soluble proteins but are rarely applied to membrane proteins at the single-molecule level, partly because of the lack of model membranes (*7*, *28*, *29*). We engineered a NompC construct with an N-terminal SpyTag (*30*), purified it, and reconstituted it into a lipid nanodisc of ~17-nm diameter containing biotin-labeled phosphatidylethanolamine (PE) (*11*, *31*). A single NompC tetrameric complex in the nanodisc was tethered between two optically trapped polystyrene beads (~2 microns in diameter) via two DNA handles: one conjugated to the N terminus of a NompC subunit and the other to the biotin-PE lipids (Fig. 1B). These ~2–kilo–base pair (kbp) DNA handles enable attachment of a single macromolecule to micron-sized beads and precise force and extension measurements (*32*). We stretched a single NompC complex by moving one optical trap relative to the other, measuring the extension and tension of the nanodisc-NompC-DNA complex.

**Fig. 2.**
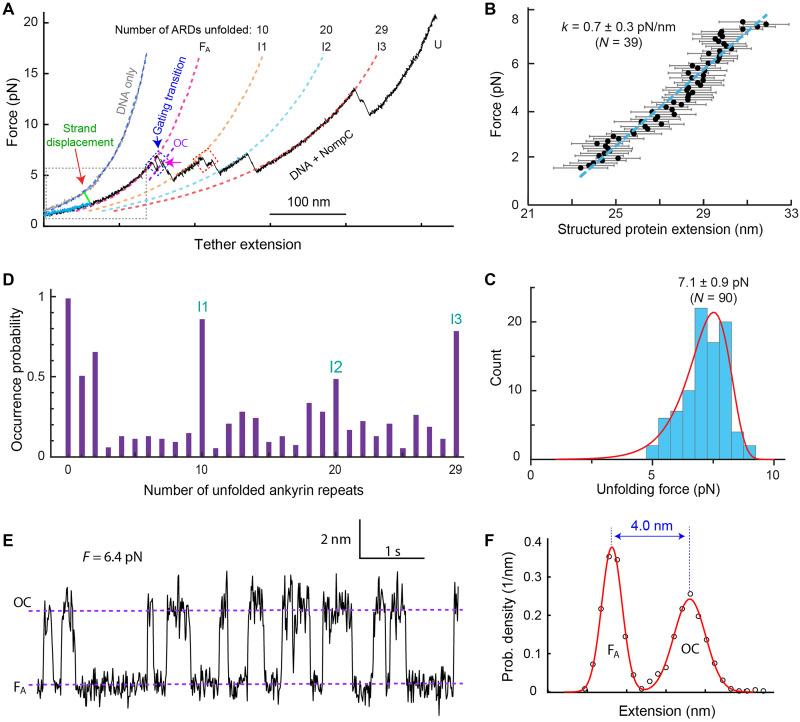
Mechanical properties of single NompC complexes. (**A**) Representative force-extension curves (FECs) of DNA handles only (gray) and nanodisc-embedded NompC tethered to DNA handles (black). States: F_A_, folded ARDs and closed channels; OC, folded ARDs and open channels; U, fully unfolded cytosolic domain; and I1-3, intermediate unfolding states. The blue dashed rectangle highlights the potential reversible gating transition [see (E)], while the magenta arrow marks initial ARD unfolding. The nanodisc-embedded NompC was inserted into the DNA handles by strand displacement (green FEC) followed by relaxation to lower force (cyan FEC). The gray dashed rectangle marks the FEC regions used to calculate the force constant of the gating spring [see (C)]. The dashed lines are best fits of different FEC regions with a worm-like chain model, showing the numbers of ARs unfolded in different major intermediates (I1, I2, I3, and U). (**B**) Force as a function of the extension of the structured NompC portion (symbol) and its linear fit (dashed line) to derive the stiffness of the protein portion. (**C**) Histogram of the force for the initial ARD unfolding (with one event marked in A) and its best model fit (red curve; see fig. S12). (**D**) State occurrence probabilities derived from the FECs of 53 individual NompC complexes tested. The three long-lived states—I1, I2, and I3—appear as three peaks. (**E**) Extension-time trajectory at a constant mean force (*F*) corresponding to the potential gating transition. The dashed lines indicate the average extensions of the native NompC state (F_A_) and the open channel state (OC). (**F**) Probability density distribution of the extension shown in (E) (circle) and its best fit with the sum of two Gaussian functions (red curve).

To determine the force constant of a single NompC complex, we needed to measure its absolute extension as a function of applied force. As the nanodisc-NompC-DNA tether between the two beads (hereafter referred to as the tether; see also fig. S1A) is primarily extended by DNA handles, we developed a method named the toehold-mediated strand displacement strategy to subtract their contributions. Although high-resolution optical tweezers offer subnanometer resolution for relative extension changes within a single tether, their resolution for absolute extensions across different tethers is typically >10 nm (*27*, *33*), due to variations in bead size and DNA attachment site (fig. S1A; see also Supplemental Text). This reduced resolution complicates the detection of protein elasticity. Our strategy overcomes this by measuring the absolute extension of a single protein using the same DNA tether (Fig. 1C and movie S1). First, we attached a DNA-only tether between two beads, joined via hybridization of two 5′-overhang DNA sequences (Fig. 1C, state 1). We pulled this tether to measure its force-extension curve (FEC; Fig. 2A, gray curve), which fit the worm-like chain model for DNA (blue dashed curve) (*34*, *35*). After relaxing the tether and holding it at low force, we introduced a NompC solution into the microfluidic channel. A single NompC complex bound to one overhang via biotin-PE in the nanodisc (Fig. 1C, state 2), and a DNA oligonucleotide attached to the SpyTag displaced the overhang via toehold-mediated strand displacement (Fig. 1C, state 3) (*36*), marked by a sudden extension decrease (Fig. 2A, red arrow). The strand displacement, combined with a control experiment (fig. S2), confirmed that a single NompC complex was pulled. This method accurately measures absolute protein extension by subtracting DNA handle extension from the total tether extension (see the “Data analysis and modeling” section). Simulations showed that our high-resolution optical tweezers can measure protein stiffnesses from 0.1 to 30 pN/nm (fig. S1), validated using a 66-nucleotide single-stranded DNA (fig. S3).

### Elasticity and unfolding of the NompC complex

When pulled up to ~7 pN, the NompC-nanodisc-DNA tether exhibited a continuous extension increase due to elastic stretching of the NompC complex and DNA handles (Fig. 2A, state F_A_). To isolate the force-dependent extension of the structured NompC portion, we calculated the N-terminal polypeptide extension using a worm-like chain model (see Materials and Methods). The nanodisc extension remained constant (~5 nm) below 7 pN, indicating high rigidity (fig. S4). Subtracting the extensions of the DNA handles, N-terminal polypeptide, and nanodisc, we derived the structured NompC extension (Fig. 2B). The NompC extension increases linearly with force, yielding a force constant of 0.7 ± 0.3 pN/nm (SEM). Thus, a NompC complex can extend ~10 nm on average when subjected to up to 7 pN force. Unlike most brittle globular proteins, which extend only a few nanometers before unfolding under force (*32*), NompC complexes are highly elastic.

At forces above ~7 pN, successive discrete extension increases or force-dependent flickering was observed (Fig. 2A), corresponding to irreversible or reversible conformational transitions, respectively. The first transition was typically reversible and is attributed to the channel’s gating transition, as detailed below. The second transition manifested as an irreversible extension jump that marks the initial ARD unfolding. The unfolding force for this event ranged from 5 to 8 pN, with a mean of 7.1 ± 0.9 pN (SD) (Fig. 2C). At forces of >15 pN, FECs of different NompC complexes overlapped with no further unfolding, suggesting complete unfolding of the cytosolic domain (state U, Fig. 2A and fig. S5). Despite their stochastic nature, the FECs showed consistent patterns across NompC complexes, revealing more than 30 unfolding intermediates (Fig. 2D and fig. S5). Three states (I1, I2, and I3) were notable for their long lifetimes (estimated by the force range of these states during pulling) and high occurrence frequency. Their extensions relative to the folded and unfolded states indicate that the NompC ARD likely unfolded stepwise from the N terminus: ~one-third (~10 ARs, I1), ~two-thirds (~20 ARs, I2), and fully unfolded (29 ARs, I3). Contour lengths from model fits confirmed these assignments (see Materials and Methods), although further validation is needed. Last, we derived the force constant of the NompC complex in I1 state, which was 0.5 ± 0.1 pN/nm (fig. S6), suggesting inter-ARD interactions. Thus, NompC ARDs unfold stepwise with distinct intermediates and elasticity.

### Refolding properties of the NompC complex

**Fig. 3.**
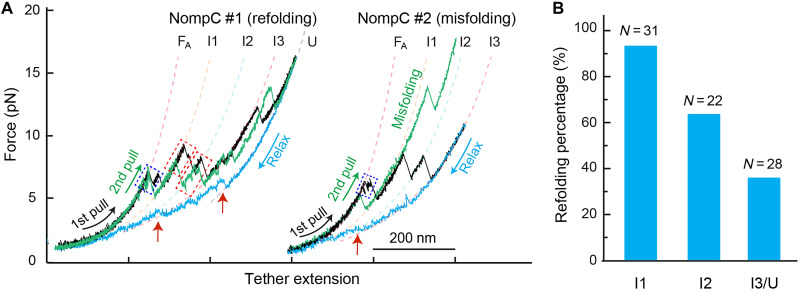
The unfolded ARDs could refold and misfold. (**A**) FECs of a single NompC complex over multiple consecutive rounds, showing NompC refolding (NompC #1) and misfolding (NompC #2). The black and green FECs are for the first and second rounds of pulling, respectively, while the cyan FECs are for relaxation. (**B**) Probabilities of successful NompC ARD refolding from the three major intermediates.

To address refolding, we relaxed the tension on an unfolded NompC subunit. Refolding began below 6 pN and completed below 1.5 pN, matching the native state extension (Fig. 3A, red arrows). This suggests that the transmembrane domain remained in the nanodisc. A pronounced hysteresis between pulling and relaxation FECs (Fig. 3A, compare cyan and black curves) implies a large energy barrier for unfolding and refolding. Repulling refolded NompC yielded an FEC overlapping the initial pull (Fig. 3A, green versus black FECs for NompC #1), confirming native state recovery. Approximately 30% of unfolded NompC complexes refolded successfully (Fig. 3B), a high fraction given NompC’s large size and complexity. Other refolded NompC complexes showed altered FECs (e.g., stability >15 pN; Fig. 3A, NompC #2, green versus black), suggesting misfolding. Refolding from less unfolded I2 and I1 intermediates showed higher probabilities (Fig. 3B), with I1 refolding at 94% (fig. S7). Thus, NompC ARDs unfold at a low force and refold efficiently from intermediates when relaxed.

### The first reversible transition as a potential gating transition

In ~60% of pulling FECs, the first intermediate reversibly transitioned with the native but ARD stretched NompC complex before ARD unfolding (Fig. 2A, between states OC and F_A_; fig. S5, blue dashed rectangles). In others, it unfolded too quickly to revisit the native state. This reversible transition was observed at constant mean force (Fig. 2E and fig. S8A), showing two-state behavior via a bimodal extension distribution fit by two Gaussians (Fig. 2F). The equilibrium force for the transition, i.e., the force with equal probability in either state, was 6.9 ± 0.6 (SD, *N* =10) pN, with an extension change of 4.7 ± 0.5 nm (fig. S8), yielding an energy change of 8.0 ± 1.1 *k*_B_*T* (4.7 ± 0.6 kcal/mol) (see Materials and Methods). We hypothesize that this represents NompC’s gating transition, supported by further evidence below.

### NompC elasticity is mainly determined by ARDs

**Fig. 4.**
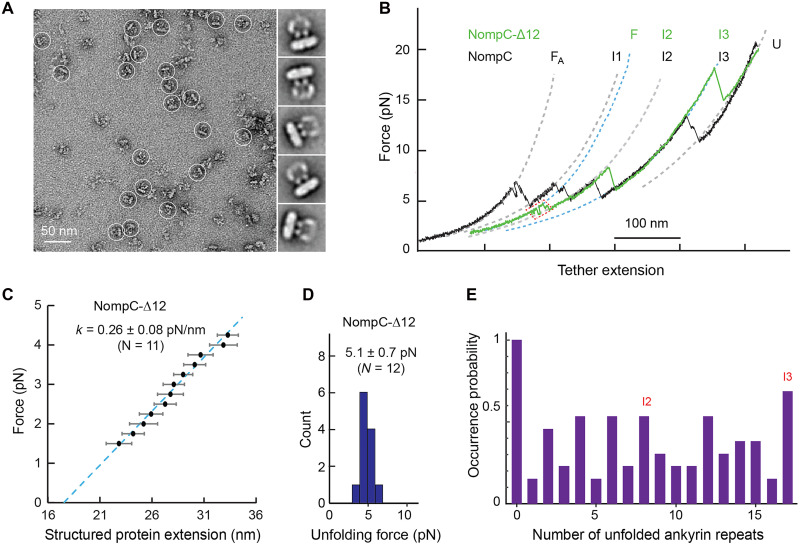
Mechanical properties of the NompC complex with the N-terminal 12 ARs removed (NompC-Δ12). (**A**) A representative raw micrograph (left) acquired by negative-stain electron microscopy (EM) and two-dimensional (2D) class average (right) of purified NompC-Δ12. Examples of NompC-Δ12 particles are highlighted with white circles in the raw micrograph. (**B**) FECs of NompC-Δ12 (green) and wild-type (WT) NompC (black) complexes. The dashed curves are best fits with the worm-like chain model. The states associated with different FEC regions are indicated: fully folded state (F_A_), intermediate states (I1, I2, and I3), and unfolded ARD state (U). The FEC for NompC-Δ12 is shifted along the *x* axis to align its unfolded state region to the corresponding region for the WT NompC complex. The two complexes have overlapping FEC regions corresponding to I3 and I2 states, corroborating our state assignments, as the N-terminal AR truncation does not significantly affect these states. (**C**) Force as a function of the extension of the structured NompC-Δ12 portion (symbol) and its linear fit (dashed line) to derive the indicated force constant of the protein. (**D**) Histogram of the force for the initial ARD unfolding event. (**E**) State occurrence probabilities derived from the FECs of 13 individual NompC-Δ12 complexes.

To assess the ARD contribution to NompC elasticity, we generated a truncation lacking the N-terminal 12 ARs or amino acids 126 to 533 (NompC-Δ12, Fig. 1B). Negative-stain electron microscopy (EM) and two-dimensional (2D) class averaging showed that the truncated NompC variant was monodisperse, properly folded, and assembled into tetramers. The overall shape and dimensions of these tetramers in 2D class averages closely resembled those of the equivalent region in full-length NompC (Fig. 4A) (*11*), despite the deletion of the distal portion of the ARDs. This observation is consistent with cooperative unfolding of the N-terminal ~10 ARs in the full-length NompC (Fig. 4B). The pulling experiment also showed a reduced force constant (0.26 ± 0.08 pN/nm versus 0.7 ± 0.3 pN, Fig. 4C). This finding indicates that ARDs significantly contribute to the measured NompC elasticity with strong interactions between ARDs from different subunits. Otherwise, shortening the ARD would increase stiffness as expected for a homogenous spring, because the spring constant is inversely proportional to the spring’s intrinsic length. In addition, the force for the initial ARD unfolding decreased from 7.1 ± 0.9 pN to 5.1 ± 0.7 pN (Fig. 4D), and the hypothesized gating transition persisted (Fig. 4B). Many FECs aligned with wild-type NompC for unfolded and intermediate states (U, I2, and I3), but I2 occurrence became diffuse due to truncation near its N-terminal boundary (Fig. 4E versus Fig. 2D), consistent with our state assignments.

### Mechanical properties of AnkB ARD

**Fig. 5.**
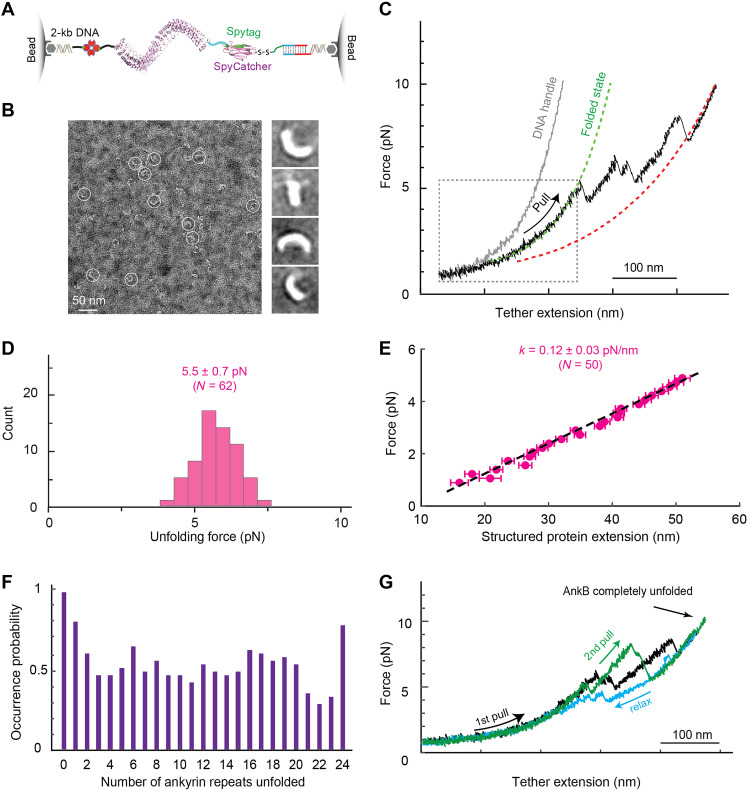
Compliant and refoldable AnkB ARD. (**A**) Schematic diagram illustrating the attachment of a single AnkB ARD to polystyrene beads using two DNA handles. (**B**) A representative raw micrograph (left) acquired by negative-stain EM and 2D class average (right) of purified AnkB ARD reveal its expected elongated helical shape. Examples of AnkB ARD particles are highlighted with white circles in the raw micrograph. (**C**) FECs obtained by pulling the DNA handle alone (gray) and the protein-DNA tether (black) using the toehold-mediated strand displacement strategy. (**D**) Histogram of the force for the first unfolding transition of AnkB ARD. (**E**) Force as a function of the extension of AnkB ARD (symbol) and its linear fit (dashed line) to derive the stiffness of the protein. (**F**) State occurrence probabilities derived from the FECs of 44 individual AnkB ARD molecules. (**G**) FECs obtained by pulling (black and green) and relaxing (cyan) a single AnkB ARD over multiple rounds, showing efficient ARD refolding.

To explore subunit interactions, we pulled a single AnkB ARD (Fig. 5A) (*16*), as isolated NompC ARDs aggregated during purification. EM confirmed AnkB ARD integrity (Fig. 5B and fig. S9). Optical tweezers revealed unfolding at 5.5 ± 0.7 pN (SD) (Fig. 5, C and D, and fig. S10) with a force constant of 0.12 ± 0.03 pN/nm (Fig. 5E), lower than that of the NompC ARD. AnkB ARD unfolded via >23 intermediates with comparable frequencies (Fig. 5F), suggesting cooperative unfolding of multiple ARs (fig. S11), and refolded with little hysteresis (Fig. 5G). Individual ARs are unstable in solution and stabilized by neighboring ARs (*37*), and most tandem ARs reversibly unfold and refold in a two-state manner (*38*). Our results are consistent with these earlier findings but differ from the studies by AFM (*20*), where individual ARs appear to unfold independently. While experimental conditions differ between optical tweezers and AFM experiments, such as stiffnesses of the force probes (~0.1 pN/nm versus >2 pN/nm) and the force loading rate used to pull single ARDs (~1 pN/s versus >100 pN/s), further investigations are required to pinpoint the differences in observations (Supplementary Text).

**Fig. 6.**
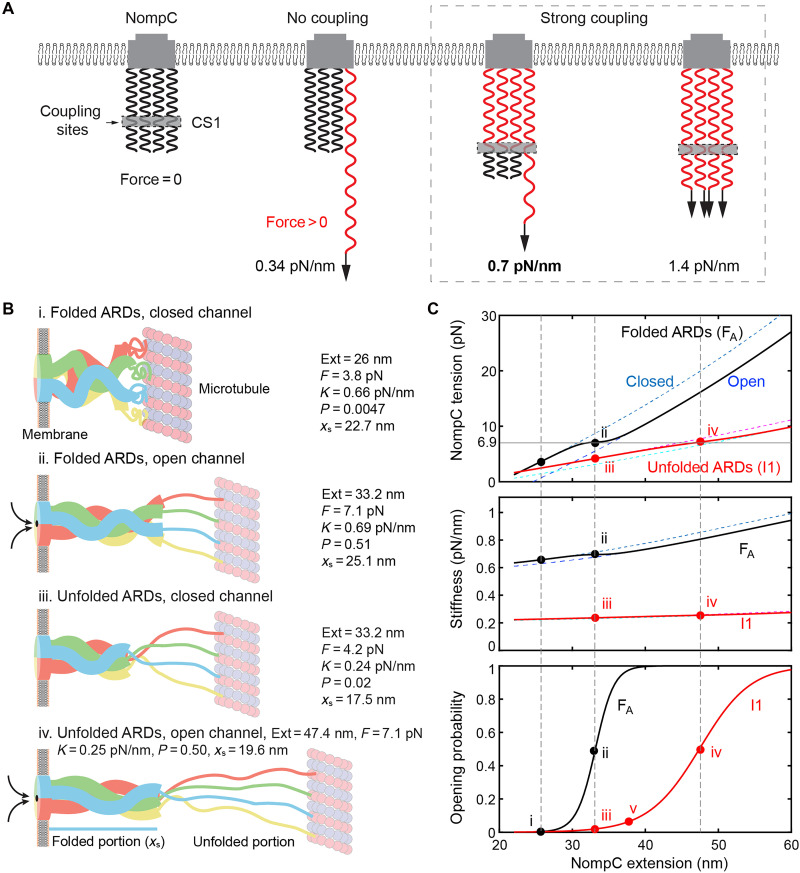
Theoretical calculations highlight the roles of ARD mechanics in modulating force-triggered channel opening. (**A**) The N-terminal contact sites (CS1) join the four ARDs into a coupled spring with an effective force constant depending on the number of ARDs being pulled. Each ARD is modeled as a uniform elastic rod with its compliance (the reciprocal of stiffness) proportional to its length. The stretched and relaxed ARD regions are shown in red and black, respectively. The stiffnesses shown were derived from the stiffness measured (in bold) by pulling a single NompC subunit (see the “Data analysis and modeling” section). (**B**) Schematics of characteristic ARD unfolding and channel states at different NompC extensions. The NompC extension (Ext), stretching force (*F*), stiffness (*K*), channel opening probability (*P*), and the extension of the structured portion (*x*_s_) associated with these states are indicated by their values, as well as their positions in (C) indicated by dots. (**C**) NompC tension (top), stiffness (middle), and channel opening probability (bottom) as a function of the NompC extension. The fully folded and partially unfolded ARD states are indicated by black and red curves, respectively, while the open and closed channel states are plotted by nearby dashed curves.

Our results show that ARDs in NompC and AnkB are compliant, unfold at low force, and refold readily. Differences in stiffness and unfolding pathways suggest strong inter-ARD interactions in the NompC tetramer (Fig. 6A). As one subunit is pulled, the lateral interactions transmit force to other subunits, likely through the two major contact sites (CS1 and CS2 in Fig. 1B) (*11*). This results in an increased force constant for the entire ARD superhelix compared to isolated individual ARDs. Truncation (NompC-Δ12) or unfolding (I1) reduced stiffness, reflecting compromised interactions.

## DISCUSSION

To assess biological significance, we modeled the tension, stiffness, and channel opening probability of the NompC tetramer (see the “Data analysis and modeling” section for details). Cryo–electron tomography analysis of NompC in fly haltere revealed that NompC complexes are overstretched to a broad range of extensions from 20 to 80 nm, with an average extension of ~43 nm, or more than double the 20-nm length of the relaxed NompC structure (*11*, *39*, *40*). To accommodate the large extensions, NompC ARDs are likely partially unfolded in vivo (*39*). The precise extent of unfolding remains unclear. For simplicity, we modeled only one partially unfolded state (I1) that retains the same gating transition as the folded state. This choice is justified because I1 readily refolds without trapping in the misfolded conformations. The folded portions of the ARDs were treated as elastic rods with a stiffness of 1.4 pN/nm (Fig. 6A), while unfolded segments were modeled as worm-like chains (*34*). Additionally, we assumed that all four subunits are tethered to the microtubule. In fully folded NompC complex, the N-terminal 123 amino acids are disordered in each subunit (Fig. 6B, states i and ii) (*9*, *11*), while the partially unfolded state contains a total of 442 disordered residues (states iii and iv). Tension in folded ARDs is higher when closed than open, increasing with NompC extension (Fig. 6C, top, cyan versus blue dashed curves). The average tension (black curve) gradually changes from the tension of the closed channel state at a low force to the tension of the open channel state at a high force, with a middle turning point at 6.9 pN. Partial ARD unfolding reduces tension (red versus black curves). Stiffness is lower than 1.4 pN/nm due to unfolded ARD portions (Fig. 6C, middle). When mechanical stimuli vary slowly with a force loading rate less than 1 pN/s used in our pulling experiments, the opening probability can be calculated on the basis of the Boltzmann distribution. With a 4.6-nm gating swing and 8 *k*_B_*T* transition energy, the opening probability is sigmoidal, with a 6.9-pN gating force (Fig. 6C, bottom). Unfolding shifts sensitivity to higher extensions, balancing it with greater dynamic range (red versus black curves). The slope of the sigmoidal distribution at a half-opening probability represents the sensitivity of NompC to detect mechanical stimuli. Thus, a folded gating spring confers a higher sensitivity but a lower dynamic range than a partially unfolded gating spring. This observation is justified by the fact that the former has significantly greater stiffness than the latter (Fig. 6C, middle).

The force-dependent kinetics of gating transition and ARD unfolding play crucial roles for NompC to sense fast mechanical stimuli such as sound. Our estimations showed that a midrange sound frequency of 1000 Hz generates a high force loading rate of ~3 × 10^3^ pN/s (fig. S12), assuming 1-nm extension change of the gating spring due to the sound vibration. Our model predicted that, under this force loading rate, the NompC channel opens at 8.7 pN force (fig. S12), slightly higher than the equilibrium force of 6.9 pN of the gating transition. In this case, NompC ARDs exhibit higher mechanical stability, which unfold at ~14 pN. These estimations demonstrate that the mechanical properties that we measured are consistent with the physiological functions of NompC.

Our model can explain several key features of NompC mechanotransduction observed in vivo. First, during strong and sustained stimuli, ARD unfolding lengthens the gating spring, thereby reducing tension at the channel and promoting channel closure (Fig. 6, A and C, from state ii to state iii). This mechanism likely underlies the rapid NompC inactivation or transient loss of sensitivity to weak stimuli that follow intense mechanical input (*41*). Second, the extended, compliant, and unfolded ARDs shifts the activation curve toward higher mechanical input, allowing the NompC channel to adapt to large displacements while remaining responsive over a broader dynamic range (*41*, *42*) (Fig. 6C, bottom). Third, upon return to weak stimuli, ARD refolding shortens and stiffens the gating spring, restoring high sensitivity to small stimuli (from state iii to state ii). ARD refolding also enables potential push-activation: a compressive stimulus that shortens the NompC complex can drive the channel from an intermediate unfolded state back to the open channel conformation (Fig. 6C, from state v to state iii to state ii), conferring bidirectional (pull or push) mechanosensitivity. More broadly, such highly dynamic gating springs may be a general requirement for tethered MSCs. By autonomously modulating stiffness and length, they function as a built-in gain control that extends the detectable stimulus range. This principle is notably paralleled by tip links in the vertebrate auditory system, which are proposed to undergo force-dependent unfolding/refolding or disruption/regeneration to achieve a similar adaptive behavior in response to sounds (*15*, *43*–*45*).

The toehold-mediated strand displacement method can generally be used in single-molecule force microscopy to improve force and extension measurements. Both optical and magnetic tweezers trap micron-sized beads as force and displacement sensors but suffer from lower resolution when measuring the absolute extension of single molecules. In addition, these beads are confined both translationally and rotationally by the traps (*46*, *47*). As a result, the pulling force can exert a torque on the beads when the attachment site of the tether is not oriented along the pulling direction, causing a systematic error in extension measurement (*47*). Our strand displacement strategy complements previous methods to minimize this error through differential measurements (*48*), which can be applied to studies of both cytosolic and membrane proteins.

Using this approach with optical tweezers, we characterized NompC’s mechanical properties. We found that ARDs in NompC and AnkB are considerably more compliant than previously estimated. Moreover, we observed ARD unfolding and refolding within a physiologically relevant force range, highlighting their likely roles in channel gating. However, whether pulling or pushing gates NompC remains debated (*9*, *49*). Molecular dynamics simulations and cell-surface experiments imply a pushing or compression force gates the NompC channel (*22*, *23*). Particularly, the pushing force generates a torque to twist open the channel. Yet, it is unclear how the torque is applied to the transduction channel without a mechanism to constrain its free rotation in the membrane. Like many elastic objects, the stiffnesses of the NompC gating spring that we measured might be independent of pushing or pulling force, especially in a low force range. However, ARD unfolding induced by pushing force, if it occurs, should be different from that caused by pulling force.

A recent study reported a stiffness of ~2 pN/nm for the NompC gating spring, close to our estimation (1.4 pN/nm), but suggested linker helices as the gating spring (*24*). Our results emphasize the notable contribution of ARDs to NompC elasticity but do not exclude the contribution from linker helices. Moreover, the mechanical properties of NompC that we derived align with those reported for the mechanotransduction channels in hair cells, including gating spring stiffness of 0.2 to 0.9 pN/nm versus 0.4 to 1.8 pN/nm (*13*), gating swing of 4.6 nm versus ~4-nm, and gating force of ~7 pN versus 3 to 35 pN (*13*–*15*, *28*). Last, gating springs in both NompC and the hearing machinery have been proposed to undergo reversible unfolding and unbinding to modulate channel kinetics (*15*, *39*). Because NompC is also involved in sound detection in some species, the comparisons suggest shared molecular mechanisms for the two tethered MSCs. Further experiments will require incorporating fluorescent ion sensors, single-channel recording or other electrophysiological measurements into the current single-molecule assay to directly dissect the gating mechanism (*50*).

## MATERIALS AND METHODS

### Expression and purification of NompC and NompC-Δ12

*Drosophila* full-length NompC (NP_001245891.1) or the truncated NompC-Δ12 was expressed and purified using the BacMam system as previously described (*11*). In brief, an N-terminal SpyTag, the gene encoding NompC, and a C-terminal 3C cleavage site with enhanced green fluorescent protein were assembled through Gibson assembly into a modified pFastBac vector with a mammalian CMV promoter. Bacmids were extracted from DH10Bac *Escherichia coli* cells after the transformation of the NompC construct. Subsequently, the bacmid was transfected into *Spodoptera frugiperda* (Sf9) cells to generate baculoviruses. The protein was expressed in human embryonic kidney 293 GnTi^−/−^ suspension cells and grown in ESF SFM Serum-Free Medium (Expression Systems LLC), supplemented with 1% fetal bovine serum (Axenia BioLogix) at 37°C. The culture was supplemented with 10 mM sodium butyrate (Sigma-Aldrich) to boost protein expression 24 hours after baculovirus infection and then further incubated at 30°C for 48 hours before harvest.

The NompC complexes were affinity purified at 4°C using green fluorescent protein (GFP) nanobody. The cell pellet from 0.5 liters of culture (~3 g) was first resuspended and lysed in 50 ml of hypotonic buffer containing 10 mM bicine (pH 8.5), 1 mM EDTA, 3 mM dithiothreitol (DTT), 1× complete protease inhibitor cocktail (Roche), and 1 mM phenylmethylsulfonyl (PMSF). The membrane fraction was collected by centrifugation at 35,000*g* for 30 min and then dispersed again by homogenization in extraction buffer [20 mM bicine (pH 8.5), 500 mM NaCl, 1 mM EDTA, 1× complete protease inhibitor cocktail, and 1 mM PMSF]. Protein was extracted in 50 ml of extraction buffer supplemented with 1% *n*-dodecyl-β-d-maltopyranoside (DDM) and 0.2% cholesteryl hemisuccinate (CHS) with gentle stirring. After a 2-hour extraction, the lysate was cleared by centrifugation at 35,000*g* for 1 hour. The supernatant was mixed with 1 ml of CNBr-activated sepharose resin (GE Healthcare) coupled with an anti-GFP nanobody (*51*) and incubated on a rotator overnight. The resin was then loaded onto a polypropylene column and washed with 10× column volume of wash buffer (WB) containing 20 mM bicine (pH 8.5), 500 mM KCl, 0.025% DDM, 0.005% CHS, 1 mM EDTA, and 3:1:1 1-palmitoyl-2-oleoyl-*sn*-glycero-3-phosphocholine:1-palmitoyl-2-oleoyl-*sn*-glycero-3-phosphoethanolamine:1-palmitoyl-2-oleoyl-*sn*-glycero-3-phospho-(1′-rac-glycerol) (0.05 mg/ml). The washed resin was incubated quiescently with 1 ml of WB containing 0.2 mg of 3C precision protease overnight to release the protein.

### Reconstituting NompC into nanodiscs

The concentration of the eluted protein was determined on the basis of its absorbance at 280 nm. Soybean polar lipid extract (Avanti) was prepared in water as described previously (*11*). For structural study by EM, the protein sample was mixed with MSP2N2 and soybean lipids at a molar ratio of NompC monomers:MSP2N2:soybean lipids = 1:2:100 (molar ratio). Additional functional lipid was added before reconstitution for optical tweezers experiments.

Biotin-PE {1,2-distearoyl-*sn*-glycero-3-phosphoethanolamine-*N*-[biotinyl(polyethylene glycol)-2000]} was incorporated into nanodiscs with a soybean lipid:biotin-PE molar ratio of 7:1. Therefore, each nanodisc contained ~60 biotin-PE lipids and multiple biotin-PE bound to the streptavidin molecule linking the nanodisc to the DNA handle (Fig. 1B). The protein-lipid mixture was placed on constant rotation until reconstitution was complete. After a 2-hour incubation, Bio-Beads SM2 (Bio-Rad) were supplemented to the mixture three times at over 3-hour intervals to initiate reconstitution by gradually removing detergents from the system. Each time, 20 ml of briefly dried Bio-Beads was added per 1 ml of protein sample used for reconstitution. One day after the last batch of Bio-Beads, the beads were removed by passing the sample through a mini-column, and the cleared sample was applied to a Superose-6 size exclusion column equilibrated with column buffer (CB) containing 20 mM bicine (pH 8.5), 500 mM NaCl and 1 mM EDTA. The peak fractions corresponding to NompC were pooled and immediately shipped at 0°C through Fed-Ex for the optical tweezer experiments.

### Expression and purification of SpyCatcher

The plasmid of SpyCatcher was generated by mutagenesis of SpyCatcher002 plasmid (purchased from Addgene with plasmid no. 102827), which added three amino acids (KCK) to the C terminus of the encoded protein sequence. The expression and purification of SpyCatcher were conducted as previously described (*52*). Briefly, the protein was transformed into *E. coli* BL21 (DE3) cells, grown in LB (Miller) at 37°C until optical density at 600 nm reached 0.6 to 0.8, and expressed by adding 0.4 mM isopropyl-β-d-thiogalactopyranoside at 30°C for 4 to 5 hours. The cells were harvested by centrifuging the culture at 5000*g*, resuspended in the lysis buffer [50 mM tris-HCl, 300 mM NaCl, and 1 mM DTT (pH 7.8)] with 1× EDTA-free protease inhibitor cocktail (cOmplete), and clarified by centrifugation at 35,000*g* for 1 hour at 4°C. The resulting supernatant was mixed with prewashed Ni–nitrilotriacetic acid (NTA) resin (Cytiva) overnight at 4°C and washed sequentially with lysis buffer supplemented with 10, 20, and 40 mM imidazole. The SpyCatcher protein was eluted from the resin in the lysis buffer containing 300 mM imidazole; dialyzed into a buffer containing 50 mM tris-HCl, 300 mM NaCl, 1 mM tris(2-carboxyethyl)phosphine (TCEP), and 5% glycerol (pH 7.8); and lastly aliquoted and stored at −80°C before use.

### Expression, purification, and biotinylation of AnkB ARD

The original plasmid of Ankyrin-B ARD was provided by Zhang’s group (*16*), which contains the gene coding for residues 28 to 872 of human AnkB (NCBI Reference Sequence: NP_001120965.1) cloned into the pDEST14 vector. The protein construct was modified by adding a Spytag003 tag (RGVPHIVMVDAYKRYK) (*52*) to the N terminus of the ARD and an Avi-tag (GLNDIFEAQKIEWHE) to the C terminus. The plasmid was transformed into *E. coli* BL21 (DE3) cells for protein expression. The purified AnkB ARD was biotinylated at the Avi-tag using the BirA biotin-protein ligase (Avidity) in the imidazole-free lysis buffer at 4°C overnight. The biotinylated protein was dialyzed in 4 liters of buffer containing 50 mM tris, 200 mM NaCl, and 1 mM EDTA (pH 7.5) to remove free biotin and DTT.

### Characterization of the structural integrity of AnkB ARD

Protein quality was examined by size exclusion chromatography through a Superdex-200 column (GE Healthcare) as well as negative-stain EM. Negative-stain EM was performed as described previously (*53*). Images were acquired on a Tecnai T12 microscope (FEI Company) operated at 120 kV and a nominal magnification of 52,000× using an UltraScan 400 camera (Gatan), corresponding to a pixel size of 2.21 Å on the specimen. 2D averages of AnkB ARD particles were analyzed by EMAN2 (*54*).

### Oligo-SpyCatcher

The oligo-SpyCatcher linked the protein to be pulled to one of DNA handles (Figs. 1B and 5A). The chemically synthesized oligonucleotide was cross-linked to the C terminus of SpyCatcher through a disulfide bridge and has the sequence:

5′-GAGGGCGTACAGTTGTATGTACGTTGGCGAGTTT – SH.

This oligo could hybridize to the DNA handle with a complementary overhang sequence. To cross-link the oligo to SpyCatcher, the oligo was first treated with 2 mM tris(2-carboxyethyl)phosphine and then buffer exchanged into a low pH Buffer A [100 mM NaH_2_PO_4_ and 400 mM NaCl (pH 5.8)] using Micro Spin-Bio chromatography columns. Next, 2 mM 2,2′-dithiodipyridine disulfide (DTDP) solution was added to the oligonucleotide solution and incubated at room temperature for 2 hours. Excess DTDP was removed by exchanging Buffer A for Buffer C [100 mM NaH_2_PO_4_ and 400 mM NaCl (pH 8.5)]. The SpyCatcher solution was similarly exchanged to Buffer C, mixed with DTDP-treated oligonucleotides with 1:50 protein-to-oligo molar ratio, and kept overnight at room temperature to allow disulfide bond formation between oligos and SpyCatcher. The oligo-labeled SpyCatcher was bound to Ni-NTA resin via the His-tag on SpyCatcher and rotated at 4°C for 2 hours; extensively washed with Buffer C; and eluted with a buffer containing 300 mM KCl, 20 mM bicine, and 300 mM imidazole (pH 8.3).

### Preparation of DNA handles

Two DNA handles, designated as left and right handles (Fig. 1C), were used in our experiments. They have the same length of 2260 base pairs and dual digoxigenin labels at one end but different overhang oligonucleotide sequences at the other end. The two DNA handles were made by polymerase chain reaction (PCR) with different templates and primer sets (ordered from Eurofins Genomics) listed below:

Left DNA handle:

Forward primer: [DIG]-ATCATCCAA-[DIG]-GGCTGAGCCT-GCAGG

Reverse primer: [Biotin]-TTTTTTTTAAGTATGTACGTTGGCGAG-**S18**-AAATCGACGCTCA AGTCAGA

Right DNA handle:

Forward primer: [DIG]-TCGCCACCA-[DIG]-TCATTTCCAGCTTTTGTG

Reverse primer: CTCGCCAACGTACATACAACTGTACGCCCTC-**S18**-ACTATCGCCACTT TTATTGGCG

Here, S18 indicates the 18-atom hexa-ethylene glycol spacer used to prevent polymerase extension to the overhang regions (underlined sequences) during PCR.

### Dual-trap high-resolution optical tweezers

Our optical tweezers system is based on a custom-made microscope, constructed on a vibration-isolation table [Technical Manufacturing Corporation (TMC), MA], within a temperature-controlled room. The optical tweezer is operated in a dual-trap mode, with one trap fixed and the other movable. Detailed specifications can be found elsewhere (*27*). In brief, a 1064-nm laser beam [continuous wave (CW), neodymium yttrium vanadate (Nd): YVO4, Spectra-Physics, 5 W) is expanded by a 5× telescope, collimated, and split (50:50) using a polarized beam splitter (PBS). The two split beams with orthogonal polarizations are reflected by a fixed mirror and a mirror mounted on a high-resolution piezoelectric actuator (Mad City Labs, WI) and combined with another PBS. The combined beams are further expanded by a 2× telescope and focused by a water-immersion, high-numerical-aperture objective (Olympus 60×, numerical aperture of 1.2) to trap two 2-μm-diameter anti-digoxigenin–coated polystyrene beads (DIGP-20-2, Spherotech Inc.) inside a microfluidic chamber (*55*). The microfluidic chamber contains three channels where the top and bottom channels flow beads to the middle channel, where they are trapped. The piezo-actuated mirror can turn along two axes and thus move one of the optical traps in the sample plane. To detect displacements of the trapped beads, the outgoing trapping beams are collected by a second identical microscope objective, split by polarization, and projected onto two position-sensitive detectors (Pacific Silicon Sensor). A light-emitting diode light source and a charge-coupled device camera are used to illuminate and view the microfluidic chamber, respectively. A LabVIEW interface is used to control and collect data from optical tweezers. To calibrate each optical trap, the Brownian motion of the trapped bead is measured. The power spectral density of the corresponding displacement trajectory is calculated and fit with a Lorentzian distribution to derive the trap stiffness and voltage-to-displacement conversion factor (*55*). The trap stiffness used in our experiments was 0.1 to 0.3 pN/nm.

### Single-molecule experiments

All pulling experiments were performed using dual-trap high-resolution optical tweezers as previously described with adaption for the toehold-mediated strand displacement strategy. Two microliters of left DNA handle (0.4 μg/μl) was mixed with 2 μl of streptavidin (1 mg/ml) and incubated at room temperature for 10 min. Then, 2 μl of 100-fold dilution of the DNA-streptavidin solution and an aliquot of 10 ng right DNA handle were each mixed with 10 μl of polystyrene beads, 2.1 μm in diameter, coated with anti-digoxigenin antibody (DIG beads, Spherotech) and incubated at room temperature for 10 min. Next, the DIG beads were diluted in 1 ml of working buffer [20 mM bicine (pH 8.5) and 300 mM KCl]. Subsequently, the two bead solutions were separately injected into the top and bottom channels of the microfluidic chamber (*55*). The central channel contained the working buffer with an oxygen scavenging system comprising glucose (10 mg/ml; Sigma-Aldrich), glucose oxidase (0.02 unit/ml; Sigma-Aldrich), and catalase (0.06 unit/ml; Sigma-Aldrich). A single left DNA handle–bound DIG bead was trapped and brought close to a single right DNA handle–bound DIG bead held in another optical trap to form a single ~4-kbp DNA tether between the two beads.

To pull the NompC complex, the nanodisc-embedded NompC complex was mixed with oligo-SpyCatcher with a 2:1 molar ratio and incubated at 4°C overnight to allow conjugation of the oligo-labeled SpyCatcher to the NompC complex. Then, the NompC complex was diluted to ~4 nM in the working buffer and flowed into the central channel, where the single DNA tether was being held at a constant force. Binding of the NompC complex to the DNA tether triggered the toehold-mediated strand displacement, leading to the attachment of the NompC complex to the two DNA handles. The flow rate was kept constant at 10 μl/min with a flow control system. AnkB ARD was pulled similarly.

The stiffness of NompC or AnkB ARD was measured by a toehold-mediated strand displacement strategy. The DNA-only tether was first pulled to measure its FEC. Then, the DNA tether was relaxed and held at around 5-pN force while waiting for protein solution to be injected into the central channel. Protein insertion into the DNA tether via strand displacement was identified by a sudden decrease in the tether extension. Last, the protein-DNA tether was pulled or relaxed to measure the corresponding FECs. All pulling and relaxation were conducted by moving one trap at a speed of 10 nm/s while the position of the other trap was kept fixed.

### Data analysis and modeling

#### Calculations of protein force constants

The time-dependent instantaneous force and extensions of the protein-DNA and DNA-only tethers during pulling were filtered by box averaging using a time window ranging from 50 to 200 ms. Then, the force data were discretized into different bins with a uniform bin size, and the extensions of the two tethers associated with each bin were averaged and subtracted. Data from different molecules (with the total number indicated by *N* in the figure) were similarly analyzed and pooled into the force-dependent bins. Last, the corresponding extensions in each bin were averaged and plotted with the SD indicated by error bars (Figs. 2B, 4C, and 5E).

#### Extension and energy of worm-like chains

We modeled the unfolded polypeptide and the DNA handle with a worm-like chain model for a semi-flexible chain (*34*). The stretching force F and the entropic energy E of the polymer chain are related to its extension x, contour length L, and persistence length P by the following formulae

$$F=\frac{k_{B}T}{P}\left[\frac{1}{4{\left(1-\frac{x}{L}\right)}^{2}}+\frac{x}{L}-\frac{1}{4}\right]$$ (1)

and

$$E_{\text{stretch}}=\frac{k_{B}T}{P}\frac{L}{4\left(1-\frac{x}{L}\right)}\left[3{\left(\frac{x}{L}\right)}^{2}-2{\left(\frac{x}{L}\right)}^{3}\right]$$ (2)

respectively. We adopted a persistence length of 40 nm for DNA and 0.6 nm for the unfolded polypeptide (*56*–*58*). The contour length of a polypeptide was calculated from its number of amino acids with 0.365 nm per amino acid. The extension of a protein $x_{P}$ consists of the contributions from its folded or structured portion $x_{s}$ and the unfolded portion $x_{u}$. The structured portion of the protein $x_{s}$ is modeled as an elastic rod with stiffness k and an intrinsic length $x_{0}$ at zero force. The extension of the unstructured portion $x_{u}$ was modeled by Eq. 1. Therefore, the extension of a single protein as a function of its tension Fcan be expressed as

$$x_{p}=x_{0}+\frac{F}{k}+x_{u}$$ (3)

The extension of a protein-DNA tether X is the sum of the extensions of the protein and the DNA handle $x_{d}$, or

$$X=x_{p}+x_{d}=x_{0}+\frac{F}{k}+x_{u}+x_{d}$$ (4)

The force-dependent extension (FEC) of the protein was calculated as the difference between the extensions of the DNA-only tethers and the protein-DNA tethers. This extension was subtracted by the extension of the unfolded portion of the protein to yield the extension of the folded protein portion. Its extension was fit with a straight line to derive the protein stiffness k and the intrinsic length $x_{0}$ (Figs. 2B, 4C, and 5E). To fit the FECs shown in Fig. 2A, the FECs of the DNA-only and protein-DNA tethers were simultaneously fit by Eqs. 1 and 4 for $x_{d}$ and X, respectively, with the persistence lengths for polypeptide and DNA and the intrinsic length and stiffness of the folded protein portion as fitting parameters.

#### Modeling of gating force and channel opening

To calculate the tension F of NompC given its extension $x_{P}$, we solved the Eq. 3 for F. The intrinsic extension of the structured NompC portion $x_{0}$varies with ARD folding and channel states. In a closed channel state, $x_{0}$ = 20 nm for the folded ARDs and $x_{0}$ = 14.5 nm for the partially unfolded ARDs, while, in an open channel state, a gating swing of 4.6 nm was added to the intrinsic extensions. The compliance (the reciprocal of stiffness) of the entire NompC gating spring is the sum of the compliances of the folded and unfolding portions of the protein, with the latter calculated from Eq. 1. The opening probability of the NompC channel was determined by the Boltzmann distribution of the closed and open states. The total energy of a stretched NompC E was the sum of the elastic energy of the folded portion of the gating spring, the entropic energy of the unfolded portion $E_{u}$, and the channel energy V, or

$$E=\frac{F^{2}}{2k}+E_{u}+V$$ (5)

where the entropic energy $E_{u}$ was calculated using Eq. 2 and F was the tension. The three terms in Eq. 5 depend on the channel state and the ARD folding state, with V=0 for the closed channel state and V=8 *k*_B_*T* (*k*_B_*T* = 4.1 pN × nm) for the open state. The channel opening probability was computed based on the Boltzmann distribution, i.e.

$$P=\frac{1}{1+\text{exp}\left(\frac{\mathrm{\Delta E}}{k_{B}T}\right)}$$ (6)

where ΔE is the energy difference of NompC in the open and closed states.

#### Estimation of the force constant of the entire NompC complex

To estimate the stiffnesses of NompC with multiple subunits being pulled (Fig. 6A), we assumed that each NompC ARD has uniform elasticity along its length and four subunits within a single NompC complex are linked at the N-terminal contact site (CS1, Fig. 1B) or the ninth AR from the N terminus. Suppose each isolated ARD with 29 ARs has a stiffness of $k_{a}$, and then the two ARD portions separated by CS1 have stiffnesses of $29k_{a}/9$ and $29k_{a}/20$ for the N-terminal portion and the C-terminal portion, respectively. The C-terminal portions of four ARDs act as four springs in parallel in response to pulling force and have an effective stiffness of $k_{C}=4\times 29k_{a}/20=29k_{a}/5$. The N-terminal portions of ARDs also act in parallel but have an effective stiffness $k_{N}$ with a spring number equal to the number of subunits being pulled (N), or $k_{N}=N\times 29k_{a}/9$. Because the N-terminal and C-terminal portions of ARDs are connected in series, the overall stiffness of the NompC complex as a function of the subunits being pulled can be calculated as

$$k_{T}\left(N\right)=\frac{k_{N}k_{C}}{k_{N}+k_{C}}=\frac{29N}{9+5N}k_{a}$$ (7)

The stiffness of an isolated ARD $k_{a}$ can be determined by the measured stiffness $k_{T}\left(1\right)$ = 0.7 pN/nm. The stiffnesses with multiple subunits being pulled are calculated using Eq. 7 as shown in Fig. 6A.

#### Estimation of the gating transition energy

The free energy of the gating transition was estimated from the reversible mechanical work required to open the channel (*58*). For each detected gating transition, this work was calculated as the product of the equilibrium force and the corresponding extension change, and the resulting values were averaged over 10 independent transitions. This estimation assumed that the gating transition occurs without substantial protein unfolding or changes in protein elasticity.

## Supplementary Materials

### The PDF file includes:

Supplemental Text Legend for movie S1 Figs. S1 to S12 References
