# Supplementary material for: Nonlinear stiffness of NompC gating spring and its implication in mechanotransduction
Source: Sci Adv. 2026 Apr 1;12(14):eaeb6165. doi: 10.1126/sciadv.aeb6165 (PMC13041742; doi:10.1126/sciadv.aeb6165)
Supplement: Supplementary file 1 — Supplemental Text Legend for movie S1 Figs. S1 to S12 References [file sciadv.aeb6165_sm.pdf]

Science Advances
AAAS

Supplementary Materials for

Nonlinear stiffness of NompC gating spring and its implication in mechanotransduction

Yukun Wang *et al.*

Corresponding author: Yongli Zhang, yongli.zhang@yale.edu

Sci. Adv. **12**, eaeb6165 (2026)
DOI: 10.1126/sciadv.aeb6165

The PDF file includes:

Supplemental Text
Legend for movie S1
Figs. S1 to S12
References

Other Supplementary Material for this manuscript includes the following:

Movie S1

Supplemental Text

Resolution to measure single protein stiffness revealed by simulations

High-resolution optical tweezers have high resolution to measure relative extension (subnanometer) and force (0.02 pN) of single tethers, but significantly lower resolution to measure absolute extension (typically >10 nm) and force (~10%) (*33*). This is because the absolute extension is limited by the uncertainty in the bead radius (Fig. S1A and its legend). Additionally, the tether may be pulled in a direction different from the moving direction of the optical trap, as trapped beads are generally not freely rotating in the trap, and the tether can attach to different sites on bead surfaces (essentially two walls to put into perspective). Our strand-displacement strategy converts absolute extension measurements on different tethers to differential extension measurements on the same DNA tether, significantly enhancing the accuracy of measuring the absolute extension of a single protein.

To explore the sensitivity of optical tweezers to measure the stiffness of a single protein molecule, we performed simulations that mimic our experimental conditions. As described in "Data analysis and modeling" in *Materials and Methods*, the NompC complex contains the structured portion, as shown by its cryo-EM structure (Fig. 1B), and the unstructured N-terminal portion. The latter is ~123 a.a. amino acids long, as further confirmed by Phyre2. We modeled the

structured portion of NompC as an elastic rod with an intrinsic length of 20 nm and a variable spring constant ranging from 0.1 to 30 pN/nm. The extensions of the unstructured polypeptide and DNA handles are characterized by worm-like chain models (*34*). For a semiflexible worm-like chain, its average extension is related to the stretching force F, the contour length L, and the persistence length P by the Marko-Siggia formulas

$$F = \frac{k_B T}{P}\left[\frac{1}{4\left(1-\frac{x}{L}\right)^2} + \frac{x}{L} - \frac{1}{4}\right]. \tag{1}$$

We chose a persistence length of 0.6 nm and a contour length of 44.9 nm (123 a.a.) for the unstructured polypeptide in NompC and a persistence length of 40 nm and a total contour length of 1,532 nm (4,507 bp) for the DNA handle.

The experimentally measured instantaneous extension of the protein-DNA tether attached between two beads fluctuates around the average extension (Fig. S1A)

$$X = x_0 + \frac{F}{k_s} + x_u + x_d, \tag{2}$$

where x_0 = 20 nm and k_s are the intrinsic length and force constant of the structured NompC and F is the tether stretching force, which is controlled by the trap separation D. x_u and x_d are the average extensions of the N-terminal unfolded polypeptide in NompC and the DNA handle, respectively, which were solved from Eq. [1] given the force F. For the DNA-only tether, $X = x_d$. The extension fluctuation is determined by Eq. [6], with the force constant k replaced by the effective force constant of the whole system, i.e.,

$$k = \frac{k_d k_p}{k_d + k_p} + k_t, \tag{3}$$

where k_d, k_p, and k_t are the force constants of the DNA handle, NompC, and optical trap, respectively. Specifically, the force constant of the whole NompC complex is $k_p = k_s k_u / (k_s + k_u)$, where k_s and k_u are the force constants of the structured and the unstructured NompC portions. The force constants of the DNA handle and the unstructured polypeptide are calculated as a derivative of the force-extension formula shown in Eq. [1], or

$$k_w = \frac{\partial F}{\partial x} = \frac{k_B T}{PL}\left[\frac{1}{2}\left(1-\frac{x}{L}\right)^{-3} + 1\right], \tag{4}$$

where the subscript $w = d$ or u represents the DNA handle or the unfolded polypeptide in NompC, respectively. Note that k_w is an implicit function of force F. For example, the force constant of the 123 a.a. polypeptide is 0.61 pN/nm at 7 pN or 0.38 pN/nm at 4 pN. Finally, the instantaneous tether extension x at a time t was sampled from a normal distribution with a mean X and standard deviation σ_x, or

$$P(x)=\frac{1}{\sqrt{2\pi}\sigma_x}e^{-\frac{(x-X)^2}{2\sigma_x^2}}. \qquad [5]$$

The extension fluctuation σ_x can be expressed as

$$\sigma_x \equiv \sqrt{\langle x^2 \rangle}=\frac{\sqrt{4k_B TB\beta}}{k}, \qquad [6]$$

where $\langle\ \rangle$ indicates thermal averaging, k_B is the Boltzmann constant, T is the temperature, B is the frequency bandwidth used in measurements, and β is the drag coefficient of the probe in the fluid (*26, 33*). For optical tweezers,

$$\beta = 6\pi\eta R, \qquad [7]$$

where η is the viscosity of the fluid, and R is the radius of the trapped bead. The instantaneous force was calculated as

$$f = F - k_t(x-X). \qquad [8]$$

Our simulations adopted the typical trap stiffness used in our experiments $k_t = 0.2$ pN/nm, the bead radius $R = 1{,}000$ nm, $k_B T$ =4.1 pN×nm, the bandwidth $B = 10$ Hz, and the viscosity of the buffer η = 1 mPa×s or 10^{-9} pN×s/nm^2.

Our simulations demonstrated that optical tweezers can measure the stiffness of single proteins within the range of 0.1 pN/nm to 30 pN/nm, encompassing the stiffness values previously measured by AFM (1.5-23 pN/nm) (*19*). We simulated force-extension curves of both the DNA-only tether and the protein-DNA tether (Fig. S1B). We derived the corresponding force-extension plots for the structured portion of the protein (Fig. S1C-F). In our differential measurement, the extension of the structured protein portion was calculated as the difference between the extensions of the protein-DNA tether and the DNA-only tether, further subtracted by the extension of the unstructured protein portion as predicted by the worm-like chain model. Then, the extensions of the structured protein portion were fit with a linear function $x = x_0\_\text{fit} + f/k_s\_\text{fit}$, yielding the best-fit force constant k_s_fit and intrinsic extension of the structured protein portion x_0_fit. These values were compared to the corresponding input values to assess the accuracy of measurements by optical tweezers.

The accuracy of the protein stiffness measurement depends significantly on the mechanical stability of the protein. In our case, the NompC complex begins to unfold at an average force of 7.1 pN. Thus, we examined the protein extension in the range of 1-7 pN with 200 data points. Given our experimental conditions - the frequency bandwidth of 10 Hz and the force loading rate of ~ 1 pN/s - this number corresponds to 3.3 pulling/relaxation cycles. Our simulation shows that the optical tweezers setup can accurately measure the elasticity of a single protein with a spring constant of 0.7 pN/nm (Fig. S1C), matching the value we measured for NompC. The simulation also recovered the input intrinsic length of the protein (20.0 nm). Similarly, proteins with spring constants of 2.0 pN/nm (Fig. S1D), 10 pN/nm (Fig. S1E), and 0.1 pN/nm were also recovered.

Expanding the force range to 1-40 pN increased the measurable protein stiffness to 30 pN/nm (Fig. S1F).

For comparison, we also simulated the protein stiffness measurement by AFM as per the previous study (*19*). In this case, no DNA handle was included (L_d=0) but the unfolded protein portion was retained. We found that, due to significant force noise ($\sigma_f = 10$ pN) of the AFM, the simulated force-extension plot for a protein with a stiffness of 0.7 pN/nm showed considerable scatter around the applied force below 7 pN (Fig. S1G). As a result, the plot could not be fit with a straight line, leading to incorrect force constant and intrinsic length of the protein. This suggests that the AFM cannot measure the stiffness of such an elastic protein. However, when the protein stiffness was increased to 1.8 pN/nm and its mechanical stability to 40 pN, AFM could recover the stiffness and intrinsic length of the protein, consistent with the previous results (*19*).

Comparisons with previous AFM studies

Our findings on the AnkB ARD differ from the previous reports in several aspects (*19*). First, the unfolding force detected by us (<10 pN, Figs. 4C, 4G, and S10) is much lower than the AFM measurement (>20 pN). Second, the force constant detected by optical tweezers (0.12 pN/nm) is significantly lower than the corresponding AFM measurement (>1.9 pN/nm). Intriguingly, another AFM experiment did not report any elastic ARD extension change before AR unfolding (*20*), although different ARDs were pulled in the two AFM experiments. Finally, the cooperative unfolding pathway derived from our experiment (Fig. S11) contrasts with the sequential unfolding pathway inferred from the AFM experiments, in which individual ARs unfolded independently (*19, 20*). In conclusion, our results on AnkB ARD quantitatively differ from the previous reports.

The different observations are partly due to different experimental conditions used in the optical tweezers and AFM experiments(*47*). First, dramatically different force loading rate was used to pull single proteins, ~1 pN/s for optical tweezers and >100 pN/s for AFM (*19, 20*). Consequently, it is possible that AFM and optical tweezers detected different protein unfolding pathways. Second, optical tweezers have significantly higher force resolution than the AFM (~0.02 pN vs ~10 pN) (*19, 26, 27*), while both have comparable extension resolution (subnanometer to nanometer). Finally, while optical tweezers allow single proteins being pulled from specific sites through the introduction of DNA handles, the AFM relies on nonspecific binding between cantilever tips and protein samples to pull single proteins, resulting in different pulling sites. The identity of the pulling site could only be inferred from the detected force-extension curves, which might introduce bias and error. Further investigations are required to pinpoint the differences in observations.

Movie S1. Cartoons showing steps of the toehold-mediated strand displacement strategy to insert a single protein into pre-stretched DNA handles for single-molecule manipulation experiments.

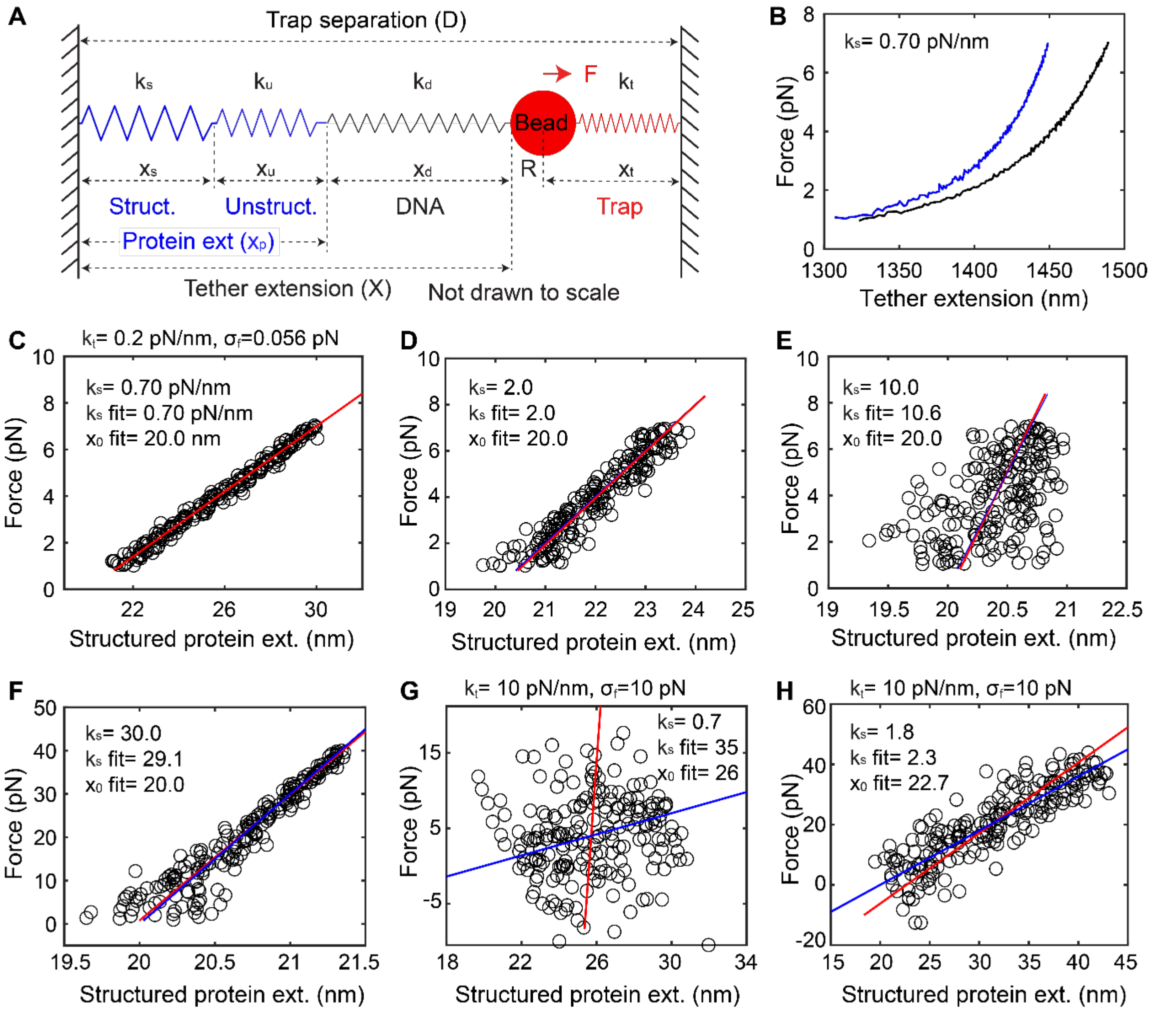

Fig. S1. Simulations show that optical tweezers with the strand-displacement strategy can measure a large range of stiffnesses (0.1-30 pN/nm) of single proteins (see Supplemental Text for details). (**A**) Schematics of the linkage between the protein, DNA handle, and beads, showing their extensions and associated spring constants. Note that the instantaneous protein-DNA tether extension is measured as $x = D - R - F/k_t$. Therefore, the absolute tether extension is subject to the error in bead radius, which varies from bead to bead by tens of nanometers. (**B**) Instantaneous force as a function of the instantaneous extension, or force-extension curves (FECs), for the DNA-only tether (blue) and the protein-DNA tether (black). The spring constant of the structured portion of the protein k_s is indicated. (**C-H**) FECs of the structured portion of the protein (symbols) with indicated spring constants k_s and their best-fits with straight lines (red). The linear fits reveal the force constants of the structured protein portion (k_s fit) and their intrinsic lengths (x_0 fit). The idealized force-extension relationships corresponding to the case of no thermal noises are shown in blue lines. Simulations were performed under experimental conditions for optical tweezers (B-F) and AFM (G-H). Different probe force constants (k_t) and force fluctuations (σ_f) were used: k_t = 0.2 pN/nm and σ_f =0.056 pN for optical tweezers and k_t = 10 pN/nm and σ_f =10 pN for AFM. The force fluctuation for optical tweezers was calculated using Eq. [6] in the Supplemental Text.

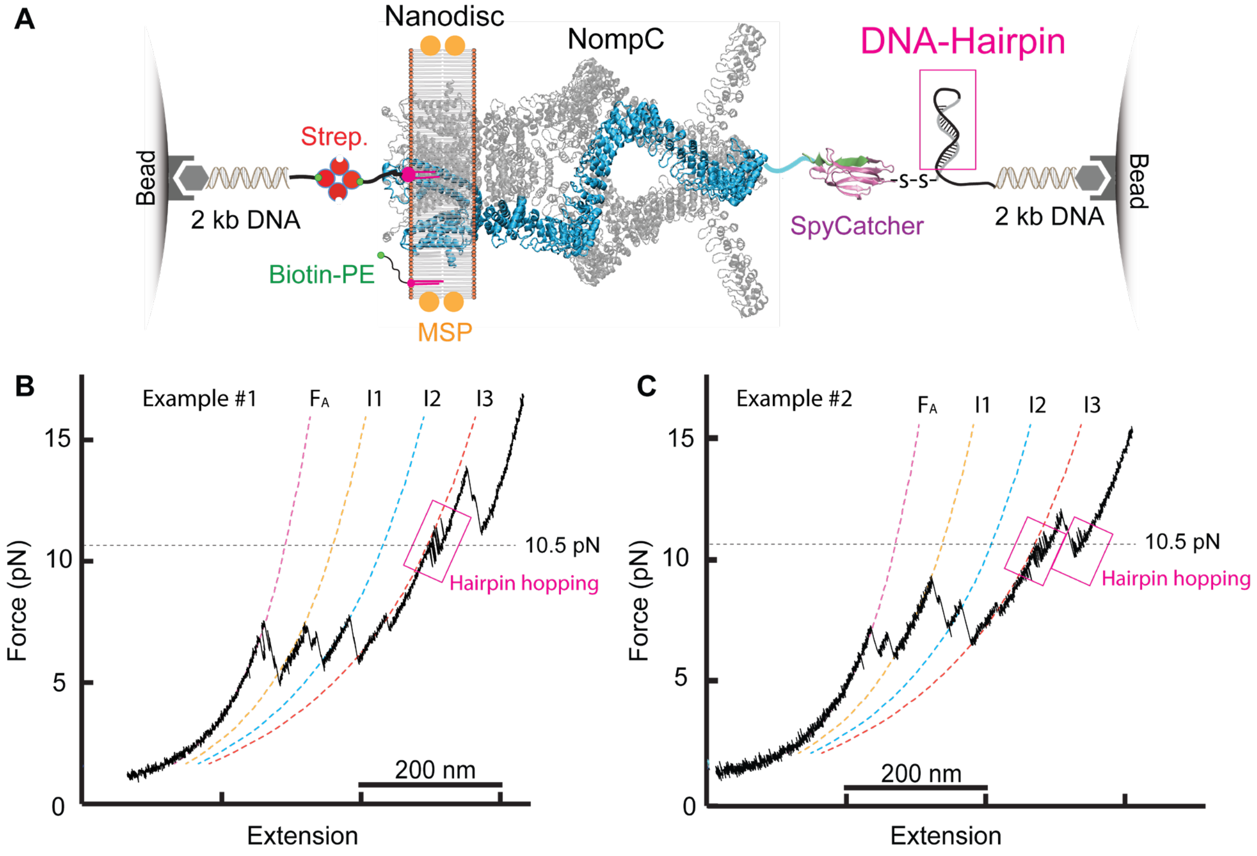

Fig. S2. Control experiment to show single NompC complexes being pulled. (**A**) A single DNA hairpin was attached to the NompC complex, whose distinct reversible unfolding at 10.5 pN helps confirm a single NompC complex is pulled. (**B-C**) Representative FECs of single NompC complexes conjugated to the DNA hairpin. The mechanical signatures (hairpin hopping) of the DNA hairpin are marked by rectangles. Note that the single DNA hairpin always hops at 10.5 pN.

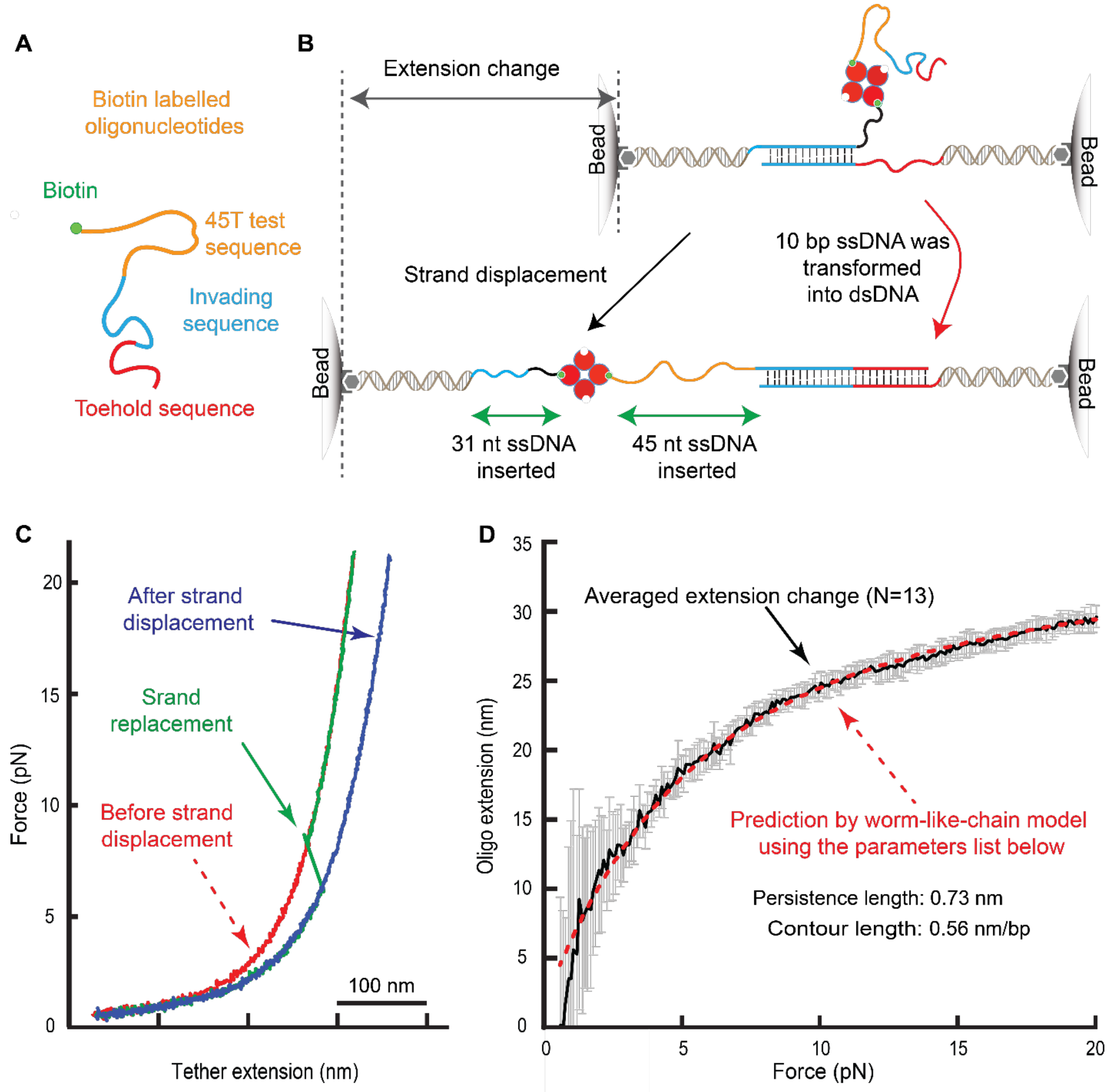

Fig. S3. Optical tweezers accurately measured the force-dependent extension of a short single-stranded DNA. (**A**) The synthesized oligonucleotide sequence contains a 5'-end biotin moiety, the 45-thymidine test sequence, the invading sequence, and the toehold sequence. (**B**) Diagram of the toehold-mediated strand displacement, which resulted in the insertion of 76-10=66 single-stranded nucleotides and 10 bp of double-stranded DNA into the pre-stretched DNA handle and associated length changes. (**C**) Force-extension curves of the DNA tether before (red) and after (blue) oligonucleotide insertion. The tether was first pulled (red), then relaxed to observe strand displacement (green), and then pulled again (blue). (**D**) Force-dependent extension of the 66-nucleotide single-stranded DNA (black curve) and its best fit with a worm-like chain model (dashed red curve) with the indicated model parameters that match the published values.

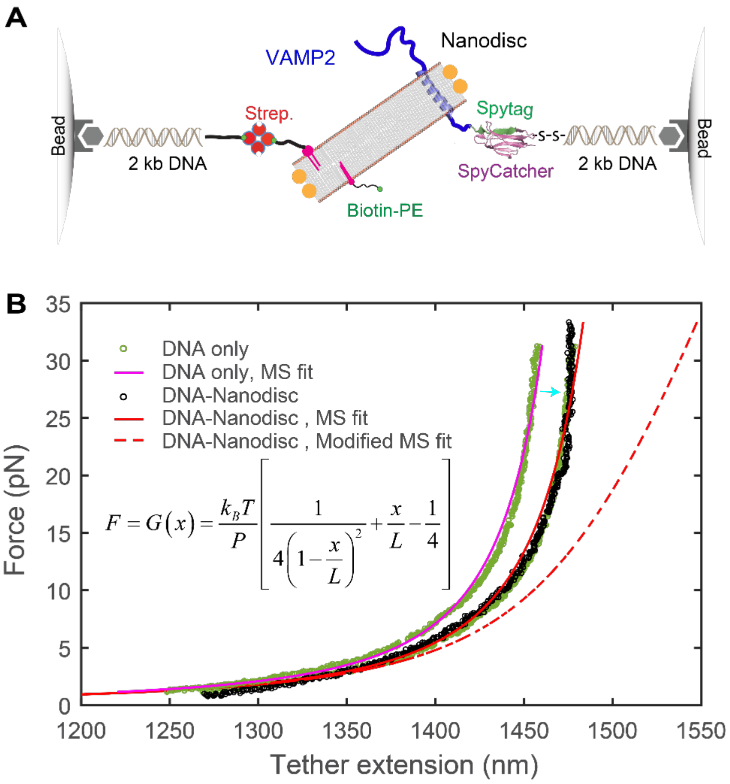

Fig. S4. The nanodisc resists pulling force with minimum deformation. (**A**) The experimental setup to pull a single nanodisc. A single SNARE protein, VAMP2, containing a single C-terminal transmembrane domain replaced the NompC complex in the nanodisc and was pulled by optical tweezers. (**B**) Force-extension curves (FECs) obtained by pulling the nanodisc (black circles) or DNA only tether (green circles). The DNA only tether refers to the two DNA handles that were hybridized in the middle without a nanodisc inserted. The FEC for DNA only tether was fit by the purple curve with the reverse function of the Marko-Siggia formula shown in the insert, or $x_d = G^{-1}(F) + x_1$ where x_d is the DNA tether extension, F is the stretching force, and x_1 is a fitting parameter used to account for the uncertainty in absolute extension measurement (Fig. S1A). Another fitting parameter is the DNA persistence length P, while the total DNA contour length is a constant, 4507 bp or 1,532 nm. The FEC for the nanodisc was fit by the red curve with the tether extension $X = x_d + x_u + \frac{F}{k} + x_2$, where x_d is the DNA handle extension already determined, x_u is the extension of the 21 a.a. unstructured polypeptide used to link VAMP2 to the DNA handle and was calculated using the reverse Marko-Siggia formula with a persistence length of 0.6 nm, k is a fitting parameter for the force constant of the nanodisc, and x_2 is another fitting parameter. The fitting yielded a force constant of 14 (±4, S.D.) pN/nm for the nanodisc. Reducing the force constant to 2 pN/nm yielded the dashed red curve. The high stiffness of the nanodisc is also supported by the fact that the two measured FECs overlap well when shifted along the x-axis (indicated by the cyan arrow).

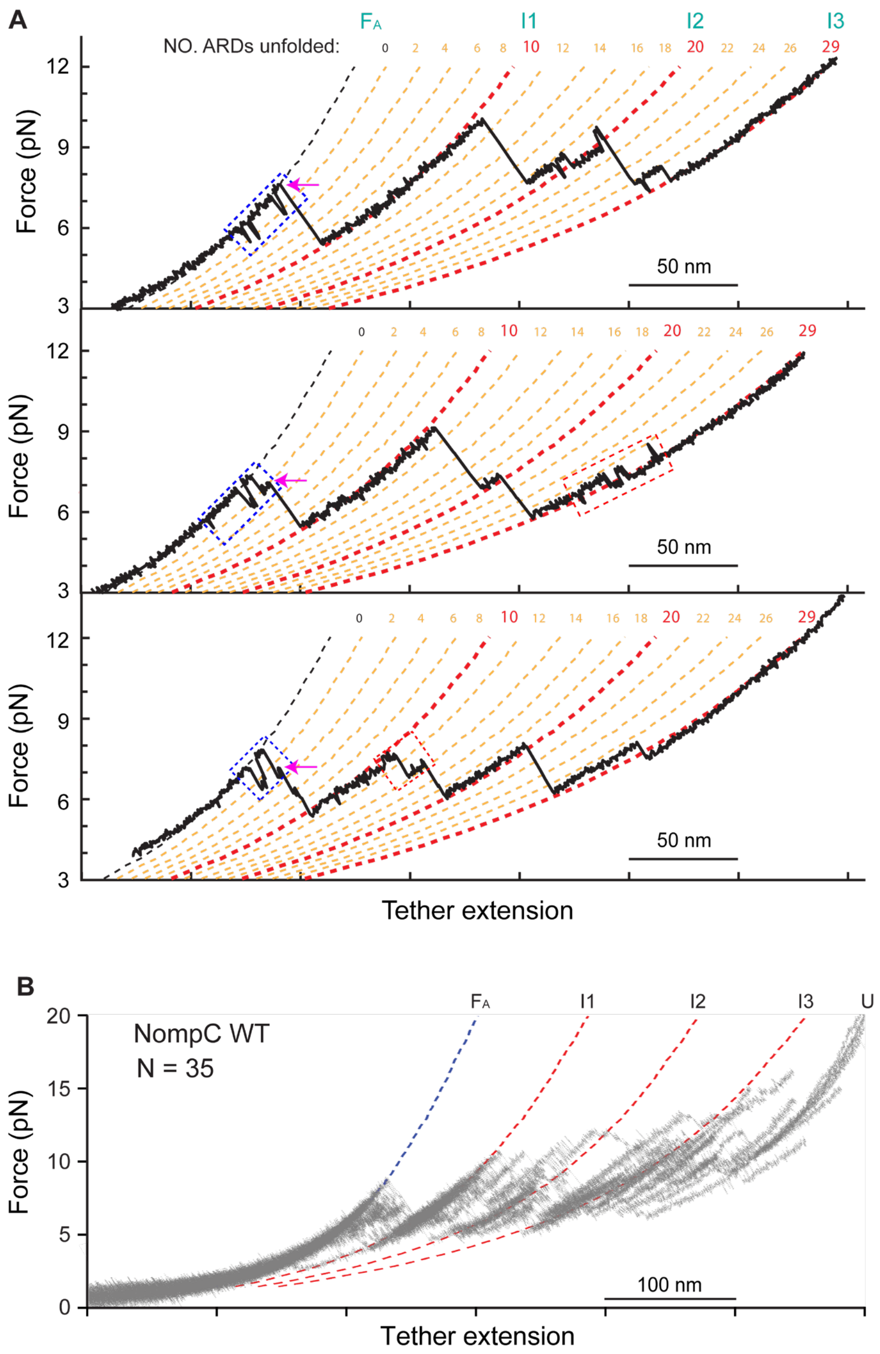

Fig. S5. FECs of different NompC complexes revealed common unfolding intermediate states with different lifetimes. (**A**) FECs (black) of three different NompC complexes and their associated long-lived intermediate states (I1, I2, and I3). The dashed curves are the best fits of the worm-like chain model to different overlapped FEC regions. The potential gating transitions with folded and partially unfolded ARD are marked by dashed blue and red rectangles, respectively. The magenta arraow mark the starting site of the first extension jump. (**B**) Overlay of FECs of 35 NompC complexes showing common unfolding intermediates.

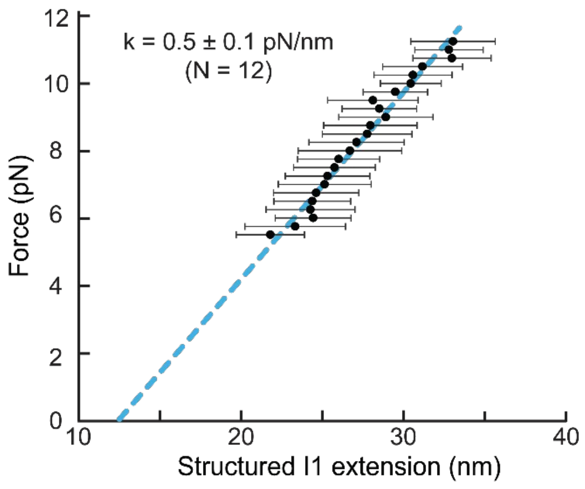

Fig. S6. **Pulling force as a function of the extension of the structured NompC with partially unfolded ARD (state I1).** Linear regression of the force-extension data revealed the force constant of the structured portion of the NompC complex, as indicated.

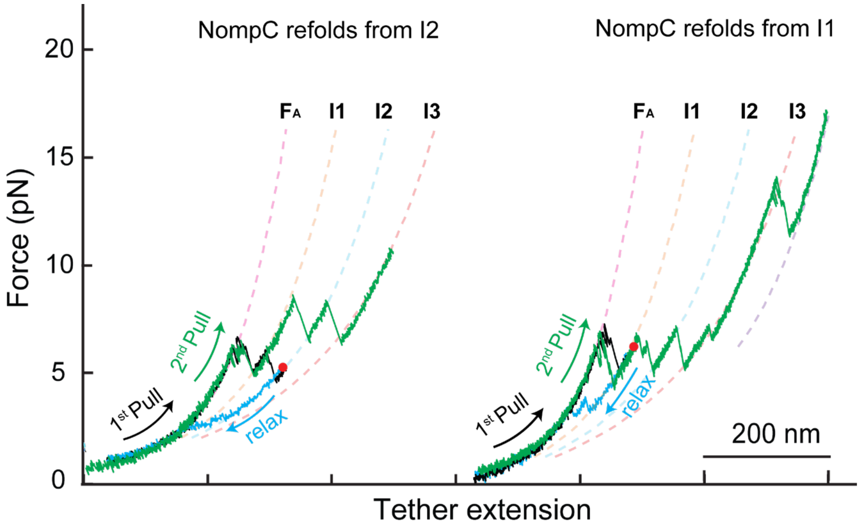

Fig. S7. FECs of NompC complexes showing refolding ARDs. Single NompC complexes were first pulled to unfold their ARDs to different intermediates (black curves) and then relaxed from these states to detect their refolding at a low force (cyan curves). The fully folded ARDs were confirmed by a second pulling round, whose FECs (green) generally overlapped the FECs from the first pulling round.

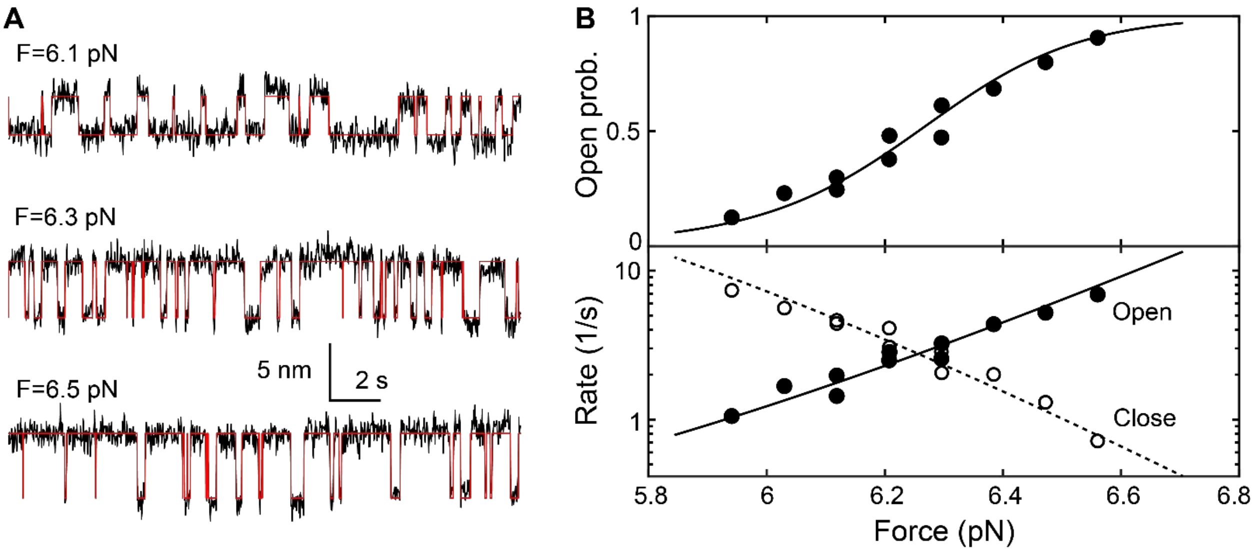

Fig. S8. **Force-dependent kinetics of the potential NompC gating transition.** (**A**) Time-dependent extension changes at indicated constant mean force (F) due to the gating transition. The red traces are idealized extension transitions derived from two-state hidden-Markov modeling (*59*). (**B**) Force-dependent opening probability (top panel) and transition rates (bottom panel) measured on a single NompC complex. Experimental measurements (symbols) were well fit by a force-dependent two-state model (*58*), yielding the best-fit curves.

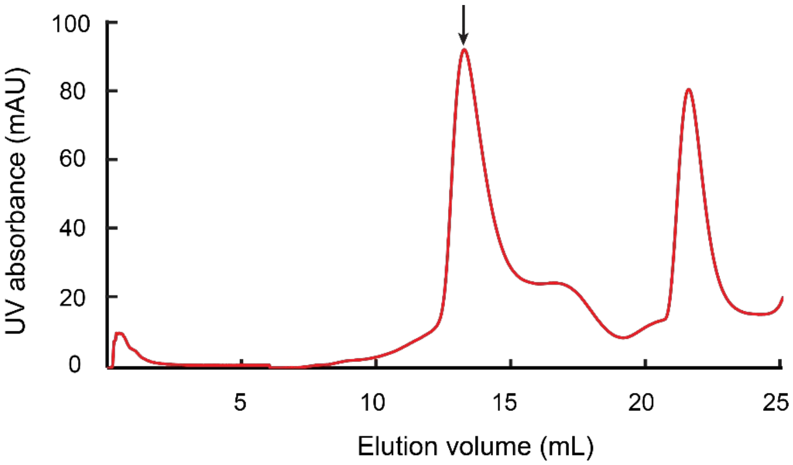

Fig. S9. **Purification of AnkB ARD. Gel filtration elution profile of the purified AnkB ARD** (peak indicated by the black arrow).

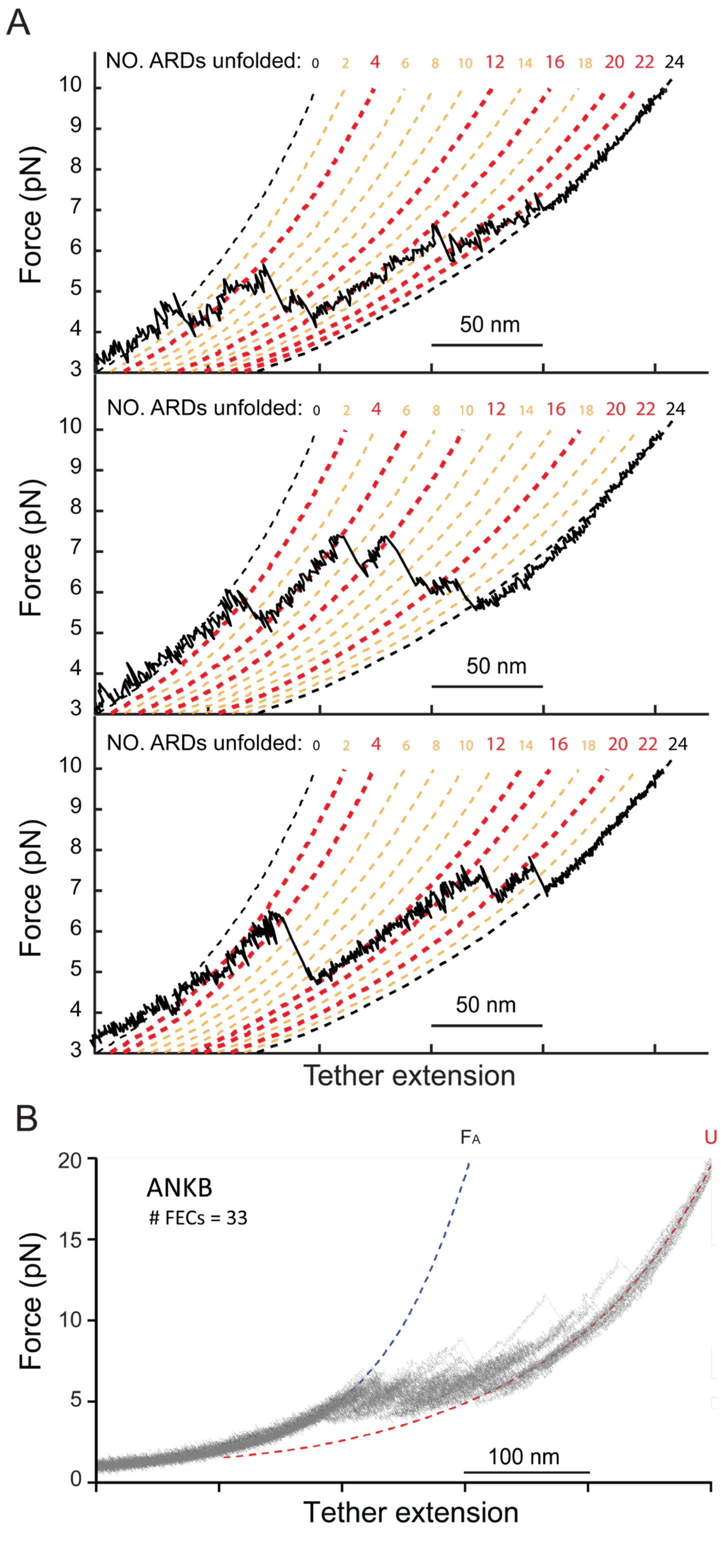

Fig. S10. **FECs showing a parallel folding pathway of AnkB ARD.** (**A**) FECs of three AnkB ARD molecules show highly parallel unfolding pathways. (**B**) Overlay of FECs from 33 different AnkB molecules.

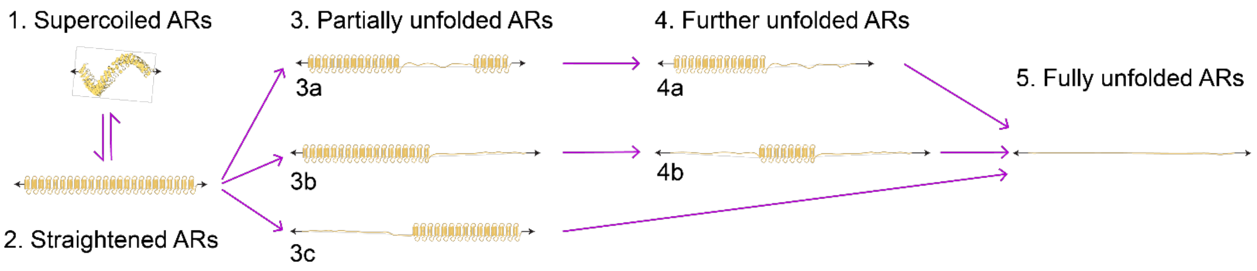

Fig. S11. Optical tweezers revealed a new ARD unfolding model. The application of force first extends the supercoiled ARD in a relaxed state (State 1) to a stretched state where the ARD is straightened with stacked ARs (State 2). Higher force breaks the stacked ARs at multiple positions in a stochastic manner, resulting in the cooperative unfolding of multiple consecutive ARs (State 3). The remaining stacked ARs further break and unfold similarly, leading to additional unfolded ARs until all ARs are unfolded at a high force (State 5). The number of substates in States 3 and 4 may vary.

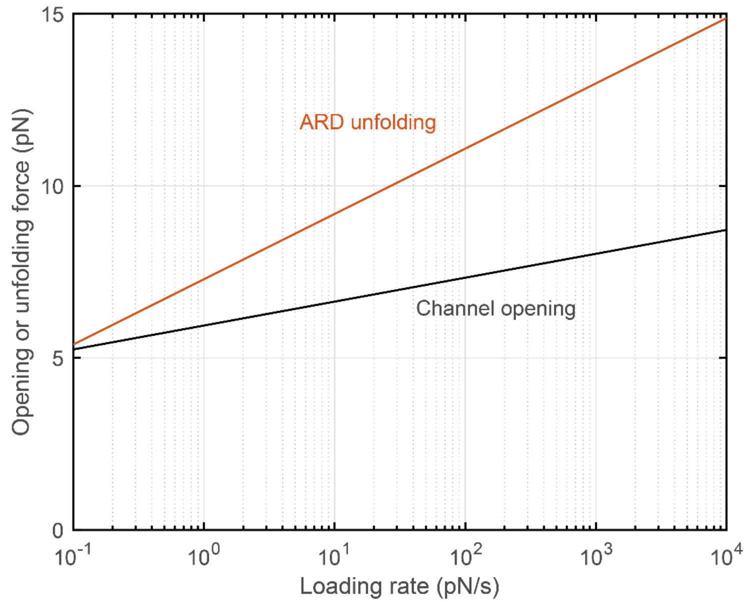

Fig. S12. Predicted most probable force for channel opening or ARD unfolding as a function of the force loading rate. The unfolding force was calculated using the formula $F^* = \frac{k_B T}{\Delta x} \ln\left[\frac{r\Delta x}{k_0 k_B T}\right]$, where Δx is the extension increase associated with the ARD conformational change from the folded state to the unfolding transition state, r is the force loading rate, and k_0 is the intrinsic unfolding rate of ARD in the absence of force (*60*). These two parameters Δx and k_0 were determined by fitting the force distribution shown in Fig. 2C to the formula $PDF(F) = \frac{k_0}{r} \exp\left[\frac{F\Delta x}{k_B T}\right] \exp\left[-\frac{k_0 k_B T}{r\Delta x}\left(e^{\frac{F\Delta x}{k_B T}} - 1\right)\right]$, which is the probability density function of the unfolding force F given a load rate r=1.25 pN/s used in our pulling experiment. The fitting yielded

the best-fit parameters: Δx= 5.4 (±0.2, std) nm and k_0=1.8 (±0.5) $\times 10^{-4}$ s^{-1}. The channel opening force F^* was calculated similarly, but with parameters derived from a model fit to the data shown in Fig. S8B.

REFERENCES

1. J. M. Kefauver, A. B. Ward, A. Patapoutian, Discoveries in structure and physiology of mechanically activated ion channels. *Nature* **587**, 567–576 (2020).

2. D. Douguet, E. Honore, Mammalian mechanoelectrical transduction: Structure and function of force-gated ion channels. *Cell* **179**, 340–354 (2019).

3. P. Jin, L. Y. Jan, Y. N. Jan, Mechanosensitive ion channels: Structural features relevant to mechanotransduction mechanisms. *Annu. Rev. Neurosci.* **43**, 207–229 (2020).

4. P. G. Gillespie, R. G. Walker, Molecular basis of mechanosensory transduction. *Nature* **413**, 194–202 (2001).

5. A. J. Hudspeth, SnapShot: Auditory transduction. *Neuron* **80**, 536–536.e1 (2013).

6. W. Zheng, J. R. Holt, The mechanosensory transduction machinery in inner ear hair cells. *Annu. Rev. Biophys.* **50**, 31–51 (2021).

7. Y. Wang, A. Kumar, H. Jin, Y. Zhang, Single-molecule manipulation of macromolecules on GUV or SUV membranes using optical tweezers. *Biophys. J.* **120**, 5454–5465 (2021).

8. R. G. Walker, A. T. Willingham, C. S. Zuker, A *Drosophila* mechanosensory transduction channel. *Science* **287**, 2229–2234 (2000).

9. W. Zhang, L. E. Cheng, M. Kittelmann, J. F. Li, M. Petkovic, T. Cheng, P. Jin, Z. H. Guo, M. C. Gopfert, L. Y. Jan, Y. N. Jan, Ankyrin repeats convey force to gate the NOMPC mechanotransduction channel. *Cell* **162**, 1391–1403 (2015).

10. Z. Q. Yan, W. Zhang, Y. He, D. Gorczyca, Y. Xiang, L. E. Cheng, S. Meltzer, L. Y. Jan, Y. N. Jan, *Drosophila* NOMPC is a mechanotransduction channel subunit for gentle-touch sensation. *Nature* **493**, 221–225 (2013).

11. P. Jin, D. Bulkley, Y. M. Guo, W. Zhang, Z. H. Guo, W. Huynh, S. P. Wu, S. Meltzer, T. Cheng, L. Y. Jan, Y. N. Jan, Y. F. Cheng, Electron cryo-microscopy structure of the mechanotransduction channel NOMPC. *Nature* **547**, 118–122 (2017).

12. D. P. Corey, A. J. Hudspeth, Kinetics of the receptor current in bullfrog saccular hair-cells. *J. Neurosci.* **3**, 962–976 (1983).

13. M. Tobin, A. Chaiyasitdhi, V. Michel, N. Michalski, P. Martin, Stiffness and tension gradients of the hair cell's tip-link complex in the mammalian cochlea. *eLife* **8**, e43473 (2019).

14. J. Howard, A. J. Hudspeth, Compliance of the hair bundle associated with gating of mechanoelectrical transduction channels in the bullfrogs saccular hair cell. *Neuron* **1**, 189–199 (1988).

15. T. F. Bartsch, F. E. Hengel, A. Oswald, G. Dionne, I. V. Chipendo, S. S. Mangat, M. El Shatanofy, L. Shapiro, U. Muller, A. J. Hudspeth, Elasticity of individual protocadherin 15 molecules implicates tip links as the gating springs for hearing. *Proc. Natl. Acad. Sci. U.S.A.* **116**, 11048–11056 (2019).

16. C. Wang, Z. Y. Wei, K. Y. Chen, F. Ye, C. Yu, V. Bennett, M. J. Zhang, Structural basis of diverse membrane target recognitions by ankyrins. *eLife* **3**, e04353 (2014).

17. J. Howard, S. Bechstedt, Hypothesis: A helix of ankyrin repeats of the NOMPIC-TRP ion channel is the gating spring of mechanoreceptors. *Curr. Biol.* **14**, R224–R226 (2004).

18. Y. Q. Tang, S. A. Lee, M. Rahman, S. A. Vanapalli, H. Lu, W. R. Schafer, Ankyrin is an intracellular tether for TMC mechanotransduction channels. *Neuron* **107**, 112–125.e10 (2020).

19. G. Lee, K. Abdi, Y. Jiang, P. Michaely, V. Bennett, P. E. Marszalek, Nanospring behaviour of ankyrin repeats. *Nature* **440**, 246–249 (2006).

20. L. W. Li, S. Wetzel, A. Pluckthun, J. M. Fernandez, Stepwise unfolding of ankyrin repeats in a single protein revealed by atomic force microscopy. *Biophys. J.* **90**, L30–L32 (2006).

21. M. Sotomayor, D. P. Corey, K. Schulten, In search of the hair-cell gating spring: Elastic properties of ankyrin and cadherin repeats. *Structure* **13**, 669–682 (2005).

22. D. Argudo, S. Capponi, N. P. Bethel, M. Grabe, A multiscale model of mechanotransduction by the ankyrin chains of the NOMPC channel. *J. Gen. Physiol.* **151**, 316–327 (2019).

23. Y. Wang, Y. F. Guo, G. L. Li, C. H. Liu, L. Wang, A. H. Zhang, Z. Q. Yan, C. Song, The push-to-open mechanism of the tethered mechanosensitive ion channel NompC. *eLife* **10**, e58388 (2021).

24. P. Hehlert, T. Effertz, R. X. Gu, B. Nadrowski, B. R. H. Geurten, D. Beutner, B. L. de Groot, M. C. Göpfert, NOMPC ion channel hinge forms a gating spring that initiates mechanosensation. *Nat. Neurosci.* **28**, 259–267 (2025).

25. E. L. Cheung, D. P. Corey, Ca^{2+} changes the force sensitivity of the hair-cell transduction channel. *Biophys. J.* **90**, 124–139 (2006).

26. C. J. Bustamante, Y. R. Chemla, S. X. Liu, M. D. Wang, Optical tweezers in single-molecule biophysics. *Nat. Rev. Methods Primers* **1**, 25 (2021).

27. G. Sirinakis, Y. X. Ren, Y. Gao, Z. Q. Xi, Y. L. Zhang, Combined versatile high-resolution optical tweezers and single-molecule fluorescence microscopy. *Rev. Sci. Instrum.* **83**, 093708 (2012).

28. A. Basu, S. Lagier, M. Vologodskaia, B. A. Fabella, A. J. Hudspeth, Direct mechanical stimulation of tip links in hair cells through DNA tethers. *eLife* **5**, e16041 (2016).

29. L. van der Sleen, J. A. Stevens, S. J. Marrink, B. Poolman, K. Tych, Probing the stability and interdomain interactions in the ABC transporter OpuA using single-molecule optical tweezers. *Cell Rep.* **43**, 114110 (2024).

30. B. Zakeri, J. O. Fierer, E. Celik, E. C. Chittock, U. Schwarz-Linek, V. T. Moy, M. Howarth, Peptide tag forming a rapid covalent bond to a protein, through engineering a bacterial adhesin. *Proc. Natl. Acad. Sci. U.S.A.* **109**, E690–E697 (2012).

31. S. G. Sligar, I. G. Denisov, Nanodiscs: A toolkit for membrane protein science. *Protein Sci.* **30**, 297–315 (2021).

32. C. Cecconi, E. A. Shank, C. Bustamante, S. Marqusee, Direct observation of the three-state folding of a single protein molecule. *Science* **309**, 2057–2060 (2005).

33. J. R. Moffitt, Y. R. Chemla, D. Izhaky, C. Bustamante, Differential detection of dual traps improves the spatial resolution of optical tweezers. *Proc. Natl. Acad. Sci. U.S.A.* **103**, 9006–9011 (2006).

34. J. F. Marko, E. D. Siggia, Stretching DNA. *Macromolecules* **28**, 8759–8770 (1995).

35. C. L. Smith, Y. Cui, C. Bustamante, Overstretching B-DNA: The elastic response of individual double-stranded and single-stranded DNA molecules. *Science* **271**, 795–799 (1996).

36. D. Y. Zhang, E. Winfree, Control of DNA strand displacement kinetics using toehold exchange. *J. Am. Chem. Soc.* **131**, 17303–17314 (2009).

37. L. K. Mosavi, D. L. Minor, Z. Y. Peng, Consensus-derived structural determinants of the ankyrin repeat motif. *Proc. Natl. Acad. Sci. U.S.A.* **99**, 16029–16034 (2002).

38. J. N. Li, A. Mahajan, M. D. Tsai, Ankyrin repeat: A unique motif mediating protein-protein interactions. *Biochemistry* **45**, 15168–15178 (2006).

39. L. D. Sun, Y. Gao, J. F. He, L. H. Cui, J. Meissner, J. M. Verbavatz, B. Li, X. Q. Feng, X. Liang, Ultrastructural organization of NompC in the mechanoreceptive organelle of *Drosophila* campaniform mechanoreceptors. *Proc. Natl. Acad. Sci. U.S.A.* **116**, 7343–7352 (2019).

40. X. Liang, J. Madrid, R. Gärtner, J. M. Verbavatz, C. Schiklenk, M. Wilsch-Bräuninger, A. Bogdanova, F. Stenger, A. Voigt, J. Howard, A NOMPC-dependent membrane-microtubule connector is a candidate for the gating spring in fly mechanoreceptors. *Curr. Biol.* **23**, 755–763 (2013).

41. A. Chadha, B. Cook, Dissection of gain control mechanisms in *Drosophila* mechanotransduction. *J. Neurosci.* **32**, 13052–13061 (2012).

42. J. Hao, P. Delmas, Multiple desensitization mechanisms of mechanotransducer channels shape firing of mechanosensory neurons. *J. Neurosci.* **30**, 13384–13395 (2010).

43. E. M. Mulhall, A. Ward, D. Yang, M. A. Koussa, D. P. Corey, W. P. Wong, Single-molecule force spectroscopy reveals the dynamic strength of the hair-cell tip-link connection. *Nat. Commun.* **12**, 849 (2021).

44. Y. D. Zhao, E. N. Yamoah, P. G. Gillespie, Regeneration of broken tip links and restoration of mechanical transduction in hair cells. *Proc. Natl. Acad. Sci. U.S.A.* **93**, 15469–15474 (1996).

45. J. Oroz, A. Galera-Prat, R. Hervas, A. Valbuena, D. Fernandez-Bravo, M. Carrion-Vazquez, Nanomechanics of tip-link cadherins. *Sci. Rep.* **9**, 13306 (2019).

46. Z. Bryant, M. D. Stone, J. Gore, S. B. Smith, N. R. Cozzarelli, C. Bustamante, Structural transitions and elasticity from torque measurements on DNA. *Nature* **424**, 338–341 (2003).

47. X. D. Zhao, X. J. Zeng, C. Lu, J. Yan, Studying the mechanical responses of proteins using magnetic tweezers. *Nanotechnology* **28**, 414002 (2017).

48. H. X. Fu, S. M. Le, H. Chen, K. Muniyappa, J. Yan, Force and ATP hydrolysis dependent regulation of RecA nucleoprotein filament by single-stranded DNA binding protein. *Nucleic Acids Res.* **41**, 924–932 (2013).

49. R. Das, L. C. Lin, F. Català-Castro, N. Malaiwong, N. Sanfeliu-Cerdán, M. Porta-De-la-Riva, A. Pidde, M. Krieg, An asymmetric mechanical code ciphers curvature-dependent proprioceptor activity. *Sci. Adv.* **7**, eabg4617 (2021).

50. B. Sakmann, E. Neher, *Single-channel recording* (Springer, ed. 2, 2009).

51. A. Kirchhofer, J. Helma, K. Schmidthals, C. Frauer, S. Cui, A. Karcher, M. Pellis, S. Muyldermans, C. S. Casas-Delucchi, M. C. Cardoso, H. Leonhardt, K.-P. Hopfner, U. Rothbauer, Modulation of protein properties in living cells using nanobodies. *Nat. Struct. Mol. Biol.* **17**, 133–138 (2009).

52. A. H. Keeble, P. Turkki, S. Stokes, I. N. A. K. Anuar, R. Rahikainen, V. P. Hytonen, M. Howarth, Approaching infinite affinity through engineering of peptide-protein interaction. *Proc. Natl. Acad. Sci. U.S.A.* **116**, 26523–26533 (2019).

53. D. S. Booth, A. Avila-Sakar, Y. Cheng, Visualizing proteins and macromolecular complexes by negative stain EM: From grid preparation to image acquisition. *J. Vis. Exp.*, e3227 (2011).

54. G. Tang, L. Peng, P. R. Baldwin, D. S. Mann, W. Jiang, I. Rees, S. J. Ludtke, EMAN2: An extensible image processing suite for electron microscopy. *J. Struct. Biol.* **157**, 38–46 (2007).

55. Y. L. Zhang, G. Sirinakis, G. Gundersen, Z. Q. Xi, Y. Gao, DNA translocation of ATP-dependent chromatin remodelling factors revealed by high-resolution optical tweezers. *Methods Enzymol.* **513**, 3–28 (2012).

56. L. Ma, A. A. Rebane, G. Yang, Z. Xi, Y. Kang, Y. Gao, Y. L. Zhang, Munc18-1-regulated stage-wise SNARE assembly underlying synaptic exocytosis. *eLife* **4**, e09580 (2015).

57. Y. Gao, S. Zorman, G. Gundersen, Z. Q. Xi, L. Ma, G. Sirinakis, J. E. Rothman, Y. L. Zhang, Single reconstituted neuronal SNARE complexes zipper in three distinct stages. *Science* **337**, 1340–1343 (2012).

58. A. A. Rebane, L. Ma, Y. L. Zhang, Structure-based derivation of protein folding intermediates and energies from optical tweezers. *Biophys. J.* **110**, 441–454 (2016).

59. Y. L. Zhang, J. Jiao, A. A. Rebane, Hidden Markov modeling with detailed balance and its application to single protein folding. *Biophys. J.* **111**, 2110–2124 (2016).

60. Y. Ren, J. Yang, B. Fujita, H. Jin, Y. Zhang, J. Berro, Force redistribution in clathrin-mediated endocytosis revealed by coiled-coil force sensors. *Sci. Adv.* **9**, eadi1535 (2023).
